# Intermembrane coupling between Bcl-xL and the IP_3_ receptor supports local Ca^2+^ transfer at ER-mitochondrial contacts

**DOI:** 10.1016/j.celrep.2026.117767

**Published:** 2026-07-30

**Authors:** Arijita Ghosh, David Weaver, Chi Li, György Hajnóczky

**Affiliations:** 1 MitoCare Center, Department of Pathology and Genomic Medicine, Thomas Jefferson University, Philadelphia, PA 19106, USA; 2 Brown Cancer Center, Department of Medicine, University of Louisville, Louisville, KY 40292, USA; 3 Lead contact

## Abstract

Bcl-xL, an anti-apoptotic Bcl-2 family protein, engages laterally with Bak/Bax in the outer mitochondrial membrane (OMM) to inhibit apoptosis and interacts with the IP_3_ receptor Ca^2+^ channels (IP3Rs) in the endoplasmic reticulum (ER) membrane to control Ca^2+^ release. It is unknown if OMM-localized Bcl-xL can also interact *in trans* with IP3Rs at ER-mitochondrial contacts to form a tethering complex that supports IP3R-mediated local Ca^2+^ transfer from ER to mitochondria. We establish that IP3R-mitochondria Ca^2+^ signal propagation depends on Bcl-xL. By targeting Bcl-xL specifically to different subcellular compartments, we find that OMM-localized Bcl-xL increases the efficacy of ER-mitochondrial Ca^2+^ transfer without changing ER Ca^2+^ release, despite attenuating mitochondrial Ca^2+^ uptake. We find interaction between Bcl-xL and each IP3R isoform occurring at the mitochondria and a complex formed by OMM-localized Bcl-xL and IP3Rs. OMM Bcl-xL interacts with IP3Rs *in trans* at ER-mitochondrial contacts to optimize local Ca^2+^ signal propagation into the mitochondria.

## In brief

Ghosh et al. report that the anti-apoptotic Bcl2 family protein, Bcl-xL, primarily found in the outer mitochondrial membrane, interacts with endoplasmic reticulum resident protein inositol 1,4,5-trisphosphate receptors. This interaction facilitates local calcium transfer at the ER-mitochondria contacts but does not impact mitochondrial calcium transfer due to global changes in cellular calcium.

## Graphical abstract

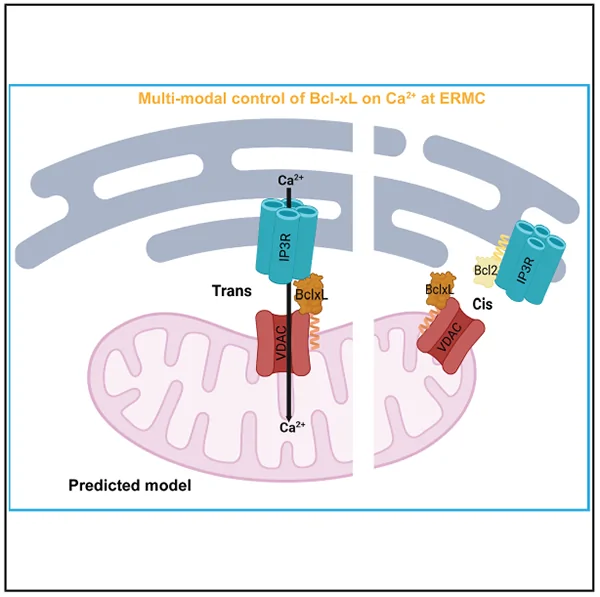

## INTRODUCTION

Mitochondria are central to many cellular processes including energy metabolism, redox signaling, and apoptosis; mitochondrial matrix [Ca^2+^] ([Ca^2+^]_m_) elevations contribute to the underlying mechanisms of all these functions. The main sources for [Ca^2+^]_m_ increases are uptake of Ca^2+^ released from the endoplasmic reticulum (ER) and Ca^2+^ entering through the plasma membrane.^1,2^

Regions of the ER membrane are closely associated with the mitochondria, forming ER-mitochondria contacts (ERMCs), which have been implicated in a wide range of mitochondrial functions including calcium signaling.^3,4^ ERMCs have been reported in many cell types and tissues, and their disruption has been linked to a broad range of diseases.^5–7^ The close membrane associations of the ERMC set the stage for local Ca^2+^ transport from ER to the mitochondria involving the ER-resident inositol 1,4,5-trisphosphate receptors (IP3Rs) and outer mitochondrial membrane (OMM) localized voltage-dependent anion channels (VDACs).^8,9^ Upon stimulation, IP_3_ binds to IP3Rs, leading to Ca^2+^ mobilization from ER into the cytosol and across the OMM via VDAC. Local micromolar [Ca^2+^] at ERMCs supports the opening of the mitochondrial calcium uniporter (mtCU) in the inner mitochondrial membrane (IMM) to relay Ca^2+^ into the mitochondrial matrix.^10,11^ The resulting matrix [Ca^2+^]_m_ rise drives ATP production, ROS generation and/or mitochondrial membrane permeabilization in synergy with other factors dictating cell fate.

IP3R channel function is modulated by a host of interacting proteins, including members of the Bcl-2 family.^12^ Bcl-2 family proteins, along with their canonical roles as apoptosis regulators, have emerged as intracellular Ca^2+^ modulators. The anti-apoptotic Bcl-2, Bcl-xL, Mcl-1, and Bcl-2 ovarian killer (BOK) have all been reported to modulate Ca^2+^ signals by interacting with IP3Rs.^13–15^ In cancer, the frequently aberrant expression of Bcl-2 proteins can influence cell survival via altered IP3R-mediated Ca^2+^ signaling and has been explored as a therapeutic target.^16–18^ Bcl-2, primarily localized to the ER, inhibits Ca^2+^ release via its BH4 domain interaction with IP3R1.^13,19,20^ However, Bcl-2-IP3R interaction is also associated with increased IP3R phosphorylation and Ca^2+^ leak.^21,22^ BOK was reported to regulate ER-mitochondria Ca^2+^ transfer because of a *cis*-interaction with IP3R in the ER.^23^ Another Bcl-2 family protein, Bcl-w, interacts with the ARM2 domain of IP3R1 via its BH4 domain, resulting in neuroprotection in sensory axons treated with a chemotherapeutic agent.^24^

Bcl-xL, such as Bcl-2, has been shown to interact with IP3Rs in multiple ways, but, unlike Bcl-2, the majority of Bcl-xL is localized to the OMM.^25^ ER-localized Bcl-xL has been shown to interact with both the N-terminal ligand-binding domain and the central modulatory domain of IP3R1 with inhibitory effects on channel function.^26^ Interaction with the C terminus of IP3R1 at two different BH3-like motifs results in sensitization of IP3R1 to IP_3_, leading to Ca^2+^ release that generates pro-survival signals promoting mitochondrial energetics.^27,28^ The same group reported that, at high expression, Bcl-xL interacts with the central coupling domain of IP3R1, leading to inhibition of IP3R.^28^ In the OMM, Bcl-xL was reported to interact with VDAC through its BH4 domain to reduce channel activity of VDAC and inhibit mitochondrial Ca^2+^ signals. Notably, these results were observed in peptide-based studies.^29^ Interestingly, the interaction of Bcl-xL with VDAC1/3 in mouse embryonic fibroblast (MEF) cells has been correlated with increased ER-mitochondria Ca^2+^ transfer.^30^

Studies with Bcl-xL targeted exclusively to the ER or OMM showed that ER-localized Bcl-xL impacted Ca^2+^ homeostasis without any effect on apoptosis, whereas maximal effect on apoptosis was observed when Bcl-xL was present at both ER and mitochondria.^31^ In a recent study, MEF cells expressing ER Bcl-xL exhibited protection against ER stress by inhibiting IP3R-mediated ER Ca^2+^ release and were more susceptible to apoptosis compared to cells expressing mitochondria-targeted Bcl-xL.^32^ Mitochondria-specific Bcl-xL was also shown to regulate mitochondrial respiration and ATP generation.^33^

Until now, the focus of research was on the lateral, intramembrane interactions of ER-localized Bcl-2/Bcl-xL with IP3R and the OMM-localized Bcl-2/Bcl-xL with VDAC1, and at both locations with the proapoptotic Bcl-2 family proteins, BAX and BAK. However, the potential of a *trans* interaction between OMM Bcl-xL and the IP3Rs to regulate local Ca^2+^ transfer from IP3Rs to the OMM at the ERMC has never been considered. There is ample evidence that Bcl-2/Bcl-xL affect both ER Ca^2+^ release and mitochondrial Ca^2+^ uptake. Moreover, sub-cellular fractionation studies have found Bcl-2 and Bcl-xL at ERMCs,^29,34^ where the ER membrane and the OMM can be within 10 nm. IP3Rs support the formation of these tight ERMCs.^8^ Several partners have been described: Grp75 binds to IP3R1/3 and VDAC1^9^ and, more recently, FUNDC1,^35^ TOM70^36^ and IRE1α^37^ were also linked to IP3R and VDAC1. Here we test the hypothesis that mitochondrial Bcl-xL and its proximity to both the IP3R and VDAC put it in an advantageous configuration to strengthen local Ca^2+^ communication between ER and mitochondria.

## RESULTS

### Bcl-xL facilitates IP3R-mediated local ER-mitochondria Ca^2+^ transfer

We postulated a trans interaction between the ER-localized IP3R and OMM-localized Bcl-xL supporting the ER-mitochondrial communication. To investigate the role of Bcl-xL in ER-mitochondrial local Ca^2+^ transfer, we first subjected WT (black) and Bcl-xLKO (gray) MEFs to carbachol (Cch) (100 μM), which stimulates IP_3_ formation through the muscarinic receptors. Muscarinic receptors, which were otherwise absent in MEFs, were introduced to the cells by co-transfection with the mitochondrial Ca^2+^ reporter. We monitored in single cells both the cytosolic ([Ca^2+^]_c_) and mitochondrial matrix ([Ca^2+^]_m_) rise tracking Fura-2 and mitochondrial matrix-targeted RCaMP (mtRCaMP) fluorescence, respectively (Figures 1A and 1B). In alignment with previous literature, on inhibition of the IP3R-mediated Ca^2+^ release by Bcl-xL,^27,31,38,39^ the [Ca^2+^]_c_ peak was higher in Bcl-xLKO (gray) than in WT cells (black, Figures 1A–1C). Despite a larger [Ca^2+^]_c_ increase in the Bcl-xLKO, the accompanying [Ca^2+^]_m_ rise was not significantly different from that in WT (Figures 1A and 1B pink traces, Figure 1D). This indicated that there could be a difference in the [Ca^2+^]_c_ signal propagation into the mitochondria between WT and Bcl-xLKO, which was difficult to ascertain accurately amidst the differences in the [Ca^2+^]_c_ increases. We reasoned that the [Ca^2+^]_c_ signal differences might reflect differences in the Cch-induced IP_3_ increases between WT and Bcl-xLKO, and Bcl-xL-mediated inhibition of the IP3R activity.

To have a level ground in terms of IP_3_ and the ensuing [Ca^2+^]_c_ in WT and Bcl-xLKO, we preloaded with caged IP_3_ (10 μM) and then subjected these cells to photolysis stimulation. Because UV photolysis interfered with Fura-2 imaging, Cal520 was used to measure [Ca^2+^]_c_ together with mtRCaMP for recording [Ca^2+^]_m_ (Figures 1E and 1F). In response to IP_3_-uncaging, similar [Ca^2+^]_c_ amplitudes (normalized to the baseline) were obtained in both WT (black) and Bcl-xLKO (gray) cells, respectively (Figures 1E–1G). At the same time, Bcl-xLKO cells showed significantly lower [Ca^2+^]_m_ elevations (Figures 1E, 1F, and 1H). As a measure of the efficacy of the ER-mitochondrial Ca^2+^ transfer, we also determined the coupling time between ER Ca^2+^ release and mitochondrial Ca^2+^ uptake. This was calculated as the time difference between the half-max of [Ca^2+^]_c_ and [Ca^2+^]_m_ peaks and was found to be longer in the absence of Bcl-xL (Figure 1I), indicating more effective Ca^2+^ signal propagation to the mitochondria in WT in the presence of Bcl-xL. As an additional measure of the mtCU activity, the instantaneous rate of [Ca^2+^]_m_ was calculated and compared for both WT and Bcl-xLKO, which revealed higher [Ca^2+^]_m_ increase flux rates of WT as opposed to Bcl-xLKO (Figure 1J). When plotted as a function of the [Ca^2+^]_c_ signal (Figure S1), the [Ca^2+^]_m_ started increasing earlier in WT cells than in Bcl-xLKO. Together, all these results point to a potential role of Bcl-xL in promoting IP_3_-induced ER-mitochondria Ca^2+^ transfer.

We next checked if the ER-mitochondrial Ca^2+^ transfer was restored in Bcl-xLKO cells rescued acutely with full-length Bcl-xL. The rescued cells were identified by expression of mtRCaMP that was co-transfected with the Bcl-xL plasmid. In acutely rescued cells (cyan), the [Ca^2+^]_c_ rise was unaltered in comparison to WT (black) and Bcl-xLKO (gray) (Figures 2A–2C, and 2D), whereas the [Ca^2+^]_m_ signal was higher in acute rescue than that in the Bcl-xLKO but still lesser than WT upon photolysis of caged IP_3_ (Figures 2A–2C and 2E). A similar difference was found for the total area under the curve for [Ca^2+^]_c_ and [Ca^2+^]_m_ traces; in the presence of similar [Ca^2+^]_c_ signals, the accompanying [Ca^2+^]_m_ signal was least in Bcl-xLKO, followed by acute rescue and WT (Figure 2G). The coupling time between ER Ca^2+^ release and mitochondrial Ca^2+^ uptake also tended toward shorter values in the Bcl-xL rescued cells (*n* = 12) than that in the Bcl-xLKO (Figure 2F). Validation of Bcl-xL expression with immunoblotting in the rescue condition revealed the appearance of the specific band that was weaker than in the WT, consistent with low efficiency of transfection (2–5%) in the MEFs (Figure 2H). Together, these results showed that acute rescue with full-length Bcl-xL can restore the ER-mitochondria Ca^2+^ transfer in the Bcl-xL-deficient cells.

### OMM-targeted Bcl-xL restores IP_3_-mediated local ERMC Ca^2+^ transfer

To test if Bcl-xL had to reside at a particular intracellular location to support ER-mitochondria Ca^2+^ transfer, we investigated what happens when Bcl-xLKO cells are substituted with differentially localized Bcl-xL, specifically cytoplasmic, devoid of a membrane targeting signal (cyto Bcl-xL, blue), ER membrane with ER targeting sequence cb5 (ER Bcl-xL, green), and OMM targeting sequence ActA conferring strict mitochondria localization (Mito Bcl-xL, burgundy) in stable rescue MEFs.^31^ Photolysis of caged IP_3_ was followed by a similar [Ca^2+^]_c_ signal among the three groups (Figures 3A–3D) but the [Ca^2+^]_m_ signal recorded in the case of both ER (green) and Mito Bcl-xL (burgundy) was significantly higher (*p* < 0.0001) than that of in cyto Bcl-xL (Figures 3A–3C and 3E). Coupling time analysis showed shorter time or effective cyto-mito coupled Ca^2+^ responses for ER and Mito Bcl-xL compared to cyto Bcl-xL (Figure 3F). [Ca^2+^]_m_ signal plotted against [Ca^2+^]_c_ signal increased earlier in the case of Mito Bcl-xL than for ER Bcl-xL, while the response in cyto Bcl-xL was the most delayed (Figure 3G). Moreover, the [Ca^2+^]_m_ peak and the [Ca^2+^]_m_ at the time of [Ca^2+^]_c_ peak were plotted as a function of [Ca^2+^]_c_ peak, which revealed higher [Ca^2+^]_m_ signals for mito Bcl-xL (burgundy) as well as for WT (black) and acute rescue (cyan) compared to Bcl-xLKO (gray) (Figure S2A). These results verify that ERMC local Ca^2+^ transfer is supported by membrane-targeted Bcl-xL, with OMM-localized Bcl-xL having an advantage in this process. The similar effect of OMM-localized Bcl-xL and ER-localized Bcl-xL on local Ca^2+^ transfer in terms of [Ca^2+^]_m_ peaks and coupling time (Figures 3E and 3F) prompted further investigation into the expression and localization of organelle-targeted Bcl-xL. An immunofluorescence (IF) experiment with the MEF cells stably expressing ER- and mitochondria-targeted Bcl-xL showed reticular localization along with mixed brighter signals around the nucleus (possibly mitochondria) in the case of ER Bcl-xL vs. well-defined mitochondrial localization of mito Bcl-xL, like the mitochondrial marker MitoBrilliant (Figure S2B). Bcl-xL immunoblotting using whole cell lysates confirmed expression of the differently targeted Bcl-xL constructs (Figure S2C). However, upon cell fractionation followed by immunoblotting, it was revealed that a significant amount of Bcl-xL was found in the mitochondrial fraction in MEF cells expressing ER-targeted Bcl-xL (Figure S2D). These results indicate that the observed [Ca^2+^]_m_ signal and coupling time in ER Bcl-xL (Figures 3E and 3F) could be contributed by the mitochondrial fraction of Bcl-xL in these cells.

### Bcl-xL is dispensable for mitochondrial Ca^2+^ uptake stimulated by global Ca^2+^ rise

Next, we checked if the lack of Bcl-xL attenuates uptake of Ca^2+^ by mitochondria from any origin. WT and Bcl-xLKO were subjected to a SERCA inhibitor, Thapsigargin (Tg, 2 μM), to deplete ER stores and then replenished with extracellular Ca^2+^ (2 mM), evoking store-operated Ca^2+^ entry (SOCE) on a global scale. Both WT and Bcl-xLKO cells showed a [Ca^2+^]_m_ rise; the Bcl-xLKO cells displayed even a significantly larger than the WT (Figures 4A–4D). Thus, Bcl-xLKO mitochondria seem to be fully competent to take up Ca^2+^ when the global [Ca^2+^]_c_ rises. A remote possibility remains that mitochondria are more closely positioned to the plasma membrane in the case of Bcl-xLKO cells. To test this, we monitored Ca^2+^ uptake in cells lacking a plasma membrane barrier next.

We assessed mitochondrial Ca^2+^ uptake in permeabilized WT and Bcl-xLKO cells upon direct exposure to Ca^2+^ bolus additions. ER Ca^2+^ uptake was inhibited by Tg and mitochondrial Ca^2+^ uptake was measured as the clearance of the extramitochondrial [Ca^2+^] with fura-FF, simultaneously with ΔѰ_m_ using TMRM (Figures 4E–4G). The Ca^2+^ clearance was abolished by the Ca^2+^ uniporter inhibitor, Ru265 (1 μM), validating mitochondrial Ca^2+^ uptake as the sole source of Ca^2+^ clearance (Figure 4E). The clearance rates were calculated for a dose of Ca^2+^ (15 nmol) increasing [Ca^2+^]_c_ to approx. 5 μM and, in the case of more Ca^2+^ (75 nmol), increasing [Ca^2+^]_c_ to approx. 20 μM. In summary, Bcl-xLKO mitochondria were more effective in Ca^2+^ clearance (Figures 4E and 4F) and showed better preservation of the ΔѰ_m_ than WT (Figure 4G). Thus, mitochondrial bioenergetics cannot explain the dependence of effective ERMC Ca^2+^ transfer on Bcl-xL.

### Interaction of Bcl-xL and IP3R in the context of OMM localization and ERMC maintenance

Having shown that Bcl-xL is required for the propagation of the IP3R-mediated [Ca^2+^]_c_ signal to the mitochondria but is not indispensable for IP3R-mediated Ca^2+^ release and mitochondrial Ca^2+^ uptake, we reasoned that Bcl-xL might help to connect IP3Rs to the mitochondria. It has also been shown previously that Bcl-xL interacts with the central modulatory region and C-terminal region of IP3R with varying affinities modulating its function^14,27,28^ but a trans interaction between OMM-localized Bcl-xL and the ER-localized IP3R has not been considered. We set out to explore the physical interaction of Bcl-xL with functionally active IP3R close to the mitochondria and to verify that if any, the interaction specifically due to gain-of-function of IP3R and not due to other off-target effects.

We rescued IP3R triple KO (TKO) HEK and HeLa cells with FLAG-tagged IP3R1 (blue Figure 5A), TKO HEK cells with IP3R2 (burgundy Figure 5B), and GFP-tagged IP3R3 (green Figure 5C) to look at their interaction with endogenous Bcl-xL by proximity ligation assay (PLA). The FLAG IP3R1 and GFP IP3R3 constructs and IP3R2 stable HEK cells were functionally validated by measuring the Cch-induced [Ca^2+^]_c_ signal (Figures 5A–5C). Expression of a mitochondria tracer, mtBFP (for both IP3R1 and IP3R3) and staining with Mito Brilliant 646 (for IP3R2) helped quantify the PLA signal specifically in the mitochondria. The PLA signal was higher in R1/R3 transfected (blue) and IP3R2 stably rescued cells (burgundy) as opposed to TKO cells (black). Moreover, much of the PLA signal came from the mitochondrial region (cyan/orange, Figures 5A–5C), indicating mitochondria-specific interaction between Bcl-xL and IP3R. The table in Figure 5D depicts the descriptive analysis of total and mitochondria-specific PLA signal in the form of number of dots per 100 μm^2^ obtained between IP3R and Bcl-xL. The antibodies used with respective fixation strategies (4% PFA in the case of R1/R3 and 100% methanol for R2) against FLAG, IP3R2, GFP, and Bcl-xL were validated by IF (Figure S4).

PLA experiments were then performed in MEFs to investigate mitochondria-specific interaction of endogenous wild-type and stably expressed organelle-targeted Bcl-xL with endogenous IP3R2/IP3R3 using Bcl-xLKO MEFs and mutant Bcl-xLK87D deficient at IP3R binding as negative controls. Figures S3A–S3C show stronger mitochondria-specific PLA signal in cells expressing mitochondria-localized Bcl-xL compared to the rest of the conditions. Antibody incompatibility in the case of IP3R1 and Bcl-xL was the reason that the interaction between them could not be checked by PLA.

We also checked the interaction between Mito Bcl-xL and IP3R1 biochemically by crosslinking followed by immunoprecipitation in MEFs stably expressing Bcl-xL localized in mitochondria (Figure 5E). IP3R1 band was detected in the crosslinked IP sample when the IP was done using the Bcl-xL antibody. However, this could not be shown in reverse order using IP3R1 antibody for the pulldown due to limitations with the antibody and detection of low amounts of endogenous IP3R1. Limitations with the IP3R2 antibody also prevented the verification of interaction between Bcl-xL and IP3R2 using chemical crosslinking. In summary, these studies provided evidence for an inter-organellar interaction formed by IP3R and Bcl-xL localized in the ER membrane and OMM, respectively.

Because IP3Rs are required to maintain the contacts between ER and mitochondria,^8^ we also asked the question if Bcl-xL deletion and the ensuing loss of IP3R-Bcl-xL inter-organellar interaction alter the closeness between ER and OMM. We found that the OMM fraction closely associated with the ER membrane decreased in Bcl-xL KO MEFs in transmission electron microscopy images, where we measured the % of mitochondrial perimeter within 0–10 nm proximity of ER segments (Figure S3D). This indicates the relevance of the Bcl-xL-IP3R coupling in ERMC structure maintenance.

### Possible interplay between mitochondrial apoptosis and local ERMC Ca^2+^ transfer

Bcl-xL-induced sensitization of the IP3R to IP_3_ through its C terminus was reported to be competitively inhibited by pro-apoptotic Bcl-2 family members Bax or truncated Bid (tBid), which is primarily produced upon death receptor engagement.^27^ We tested if tBid can suppress the Bcl-xL-mediated enhancement of the IP3R-mitochondria Ca^2+^ transfer in Bax Bak double knockout (DKO) MEF cells, in which tBid does not induce apoptosis (Figure S4). Our hypothesis was that in the presence of tBid (a known BH3-binding interactor of Bcl2/Bcl-xL) Bcl-xL will be unavailable to bind IP3R and to exert its effect on ER-mitochondria Ca^2+^ transfer. Upon tBid overexpression, there were some hints of less efficient IP_3_-induced local Ca^2+^ transfer with longer coupling time and equal [Ca^2+^]_m_ signal as control cells despite a higher [Ca^2+^]_c_ signal (Figures S5A–S5D and 5G). However, the results did not show significant suppression of the [Ca^2+^]_m_ signal, which might have been partly because of the very low transfection efficiency in DKO MEFs (Figure S5I). Thus, further studies will be needed to validate if ERMC Ca^2+^ transfer might be negatively affected by pro-apoptotic stimuli via limiting the ability of OMM Bcl-xL to bind to the IP3R.

## DISCUSSION

Anti-apoptotic Bcl-2 family proteins have been historically implicated in cell survival, with ER and mitochondrial calcium being an important component of that process. Bcl-xL so far has been highlighted as a cis interactor and modulator of IP3R and VDAC individually. We found that Bcl-xL, localized in the OMM, also interacts with the IP3R, supports the ERMC, and promotes [Ca^2+^]_c_ signal propagation from the IP3R to the mitochondria. This is due to an enhanced local Ca^2+^ transfer because neither ER Ca^2+^ release nor mitochondrial Ca^2+^ uptake is intrinsically stimulated. Thus, our work uncovers a new role for mitochondrial Bcl-xL in in-terorganellar contact site formation, which might have relevance in fundamental ERMC functions beyond local calcium signaling.

Upon stimulation of the cells with an IP_3_-linked agonist, we documented an enhanced [Ca^2+^]_c_ increase in Bcl-xLKO cells. However, no significant difference between WT and Bcl-xLKO cells occurred when endogenous IP_3_ production was bypassed, and IP_3_ was directly presented by uncaging. We aimed to stimulate the IP3Rs by a saturating dose of IP_3_ because the inhibitory/stimulatory effects of Bcl-2/Bcl-xL on IP3R activity were confined to suboptimal stimulation, and maximal IP3R activation was not sensitive to Bcl-xL, consistent with previous studies.^27,30,38^ Still, it remains possible that opposing effects of Bcl-xL on ER Ca^2+^ store loading and on various binding sites of the IP3R Ca^2+^ channel^14,27,28,39^ balanced out each other to produce similar [Ca^2+^]_c_ increases in WT and Bcl-xLKO cells, at least when maximal IP_3_ was directly presented. In any case, ER Ca^2+^ release seemed to be unaffected by the lack of Bcl-xL, enabling us to specifically examine the ER-mitochondrial Ca^2+^ transfer and the resulting mitochondrial Ca^2+^ uptake.

While the maximal IP_3_-induced ER Ca^2+^ release was unaffected in the Bcl-xLKO cells, the corresponding [Ca^2+^]_m_ rise was decreased and was normalized upon acute rescue of Bcl-xL. Thus, mitochondria likely accumulated less effectively the Ca^2+^ presented by the IP3R in the absence of Bcl-xL. This could have happened because the mitochondrial Ca^2+^ uptake mechanism (VDACs and mtCU) or the driving force of Ca^2+^ uptake (mostly ΔѰ_m_) was frail in the absence of Bcl-xL but our studies of the [Ca^2+^]_m_ signal upon global Ca^2+^ increases during SOCE and mitochondrial Ca^2+^ clearance of directly presented Ca^2+^ showed unaffected or enhanced response when Bcl-xL was absent. Furthermore, the ΔѰ_m_ was also unattenuated in Bcl-xLKO. The most likely alternative explanation for the weakened IP_3_-linked [Ca^2+^]_m_ signaling was that the local transfer of Ca^2+^ from IP3R to the mitochondrial Ca^2+^ uptake sites was ineffective in the absence of Bcl-xL. In summary, though Bcl-xL does not enhance the Ca^2+^ release by maximally activated IP3Rs and acts as a suppressor of mitochondrial uptake, it quickens and enhances the mitochondrial response to IP3R-mediated Ca^2+^ release, providing evidence for Bcl-xL’s role in local Ca^2+^ transfer at ERMC. It has been shown before that there is no sealed Ca^2+^ tunnel between ER and mitochondria^40^ but a physical interaction of IP3R with OMM proteins might create an effective and short path for Ca^2+^ transfer. Originally, IP3R1 was described to bind VDAC with the assistance of GRP75^9^ but later several IP3R binding partners were proposed in the OMM, including TOM70,^36^ FUNDC1,^35^ Sigma 1 receptor,^41^ DJ1,^42^ transglutaminase type 2,^43^ IRE1a,^37^ and a-synuclein.^44^ However, OMM Bcl-xL has never been considered as a binding partner.

Previously, the effects of Bcl-xL on IP3R and ER Ca^2+^ were attributed to ER membrane-localized Bcl-xL, but Bcl-xL has preferential distribution in the OMM.^25,45^ Bcl-xL in the OMM was described to interact with VDAC to attenuate Ca^2+^ passage^46^ and staurosporine-induced apoptosis-related Ca^2+^ transfer to the mitochondria.^29^ Differently, Bcl-xL-VDAC interaction was also linked to effective mitochondrial Ca^2+^ transfer in response to agonist stimulation of intact MEFs and direct Ca^2+^ exposure of permeabilized MEFs.^30^ However, we did not see a reduced mitochondrial Ca^2+^ uptake in permeabilized Bcl-xLKO cells when exposed to either low or high doses of Ca^2+^. In the light of these results and considering that the IP3R-mitochondrial Ca^2+^ transfer takes place where the ER and mitochondria are tightly apposed, we reasoned that OMM Bcl-xL might engage the IP3Rs in intermembrane interaction (see speculative scheme in Figure 6). This idea gained support when we rescued Bcl-xLKO cells with cytosol-, ER membrane- and OMM-targeted Bcl-xL constructs, respectively, and found that the OMM-targeted Bcl-xL supported the IP_3_-induced [Ca^2+^]_m_ signal most effectively. Notably, ER membrane-targeted Bcl-xL also had a positive outcome on the [Ca^2+^]_m_ signal, which is likely a result of the fraction of Bcl-xL ending up in the mitochondria, as found in both immunocytochemistry and cell fractionation results (Figure S2C). Alternatively, it might also be because of an effect on ER Ca^2+^ transport or because ER Bcl-xL can bridge to an unidentified OMM protein. Further evidence for OMM Bcl-xL-IP3R interaction came from the co-immunoprecipitation studies and from the PLA studies where Bcl-xL displayed the most interaction with each IP3R isoform in the mitochondria. PLA with organelle-targeted Bcl-xL showed enhanced mitochondria-specific interaction between IP3R2 and mito Bcl-xL compared to ER Bcl-xL. Collectively, these findings provide considerable support for OMM Bcl-xL forming a trans interaction with the IP3R to promote ER-mitochondrial local Ca^2+^ transfer.

Based on the 3D structural models of IP3R that have been published,^47,48^ the sheer size of the tetrameric structure would limit the possible interaction sites of Bcl-xL bound to either the OMM or ER membrane. The last helix of the C-terminal domain (C4) has been proposed to bind Bcl-xL in a similar manner as the BH3 helix of pro-apoptotic Bcl-2 family proteins.^28^ Considering the structural constraints, this interaction seems much more feasible in a trans configuration (Figure S6A), though from the extant models that resolve this helix, it is not clear that the putatively important residues are, in fact, accessible. Using Alphafold2 multimer,^49^ we found a possible interaction between the final, C-terminal flexible domain and the BH3-binding pocket of Bcl-xL (Figure S6B).^50^ A possible arrangement for the protein-protein interactions is that the Bcl-xL molecule that has a trans interaction with the IP3R also has a cis interaction with VDAC, creating a ternary complex that brings the Ca^2+^ source and Ca^2+^ acceptor of the OMM into close connection with each other (see speculative scheme in Figure 6).

Based on our electron micrographs showing decreased presence of tight ERMC upon Bcl-xL deletion, the Bcl-xL-dependent mechanism might contribute to tethering OMM to the ER. Trans interactions of Bcl-xL at ERMC might support a range of functions that depend on close associations of the ER and OMM. Our findings highlight in this regard the potential relevance of OMM Bcl-xL. One can speculate if ER Bcl-xL also reaches out to OMM partners. Notably, for Bcl-xL to have an inhibitory effect on apoptosis, ER targeting failed, and mitochondrial localization of Bcl-xL was required.^31^ This outcome is not surprising because apoptotic OMM permeabilization mediated, for example, by Bak and Bax is unlikely to be confined to the ERMC regions of the OMM only. However, more investigation is needed to discern the connection, or the lack of it, between Bcl-xL’s role in ER-mitochondrial Ca^2+^ transfer and apoptosis inhibition. With the ERMCs being implicated in multifarious cancers and a history of Bcl2 family proteins explored in cancer therapeutics, it would be interesting to further study the implications of the effect of Bcl-xL in ER-mitochondria local Ca^2+^ transfer in the context of apoptosis and cancer progression as well as therapeutics.

### Limitations of the study

Low transfection efficiency of the MEF models used posed a limitation in evaluating the effect of Bcl-xL in acute rescue conditions, which was dealt with by stably rescuing MEFs expressing organelle-targeted Bcl-xL. However, it is of note that the passages for these stably rescued cells are different from those of the WT and Bcl-xLKO MEFs. Also, low transfection efficiency of Bax-Bak DKO MEFs posed a limitation to compare competitive binding between Bcl-xL and tBid with IP3R. Lack of IP3R isoform-specific dependence information in crosslinking studies limits insights into interaction between IP3R and Bcl-xL, although we tried to incorporate all three IP3R isoforms in combined PLA studies to compensate for this. The modeling studies were done only using IP3R1 and can be potentially strengthened with inclusion of other isoforms. The modeling prediction was not tested experimentally e.g., by using truncated or mutant IP3R1, which can be an interesting future extension of the study.

## RESOURCE AVAILABILITY

### Lead contact

Requests for further information, resources and reagents should be directed to and will be fulfilled by the lead contact, György Hajnó czky (gyorgy.hajnoczky@jefferson.edu).

### Materials availability

No new materials have been generated in this study.

## STAR★METHODS

### EXPERIMENTAL MODEL AND STUDY PARTICIPANT DETAILS

#### Cells

Wild-type (WT) and IP3R-deficient (TKO) HeLa cells were provided by Katsuhiko Mikoshiba (RIKEN Brain Science Institute, Saitama, Japan). Wild-type (WT) and IP3R-deficient (TKO) HEK cells were procured from David Yule.^51^ Wild-type (WT), Bcl-xL-deficient (Bcl-xLKO), cytoplasm, mitochondria and ER targeted Bcl-xL expressing stable mouse embryonic fibroblasts (MEFs) were obtained from Chi Li and generated as previously described.^31^ Cells were cultured in Dulbecco’s Modified Eagle’s medium (DMEM), with 4.5g/L glucose (Gibco, Cat# 11965118) supplemented with 10% FBS, 2mM glutamine (Invitrogen), 100 units/ml penicillin/streptomycin (Lonza^™^, Biowhittaker^™^) in 5% CO2 and 95% air at 37°C. Cell line authentication was not performed. All cells were periodically checked for Mycoplasma contamination and they tested negative.

### METHOD DETAILS

#### Transfection and transduction

Cells were transfected using Lipofectamine 3000 (Thermo Fisher) with 1-2mg total DNA according to the manufacturer’s protocol. WT, Bcl-xLKO MEFs were transfected with muscarinic receptor and matrix targeted RCaMP for simultaneous cytoplasmic and mitochondrial Ca^2+^ measurements. Bcl-xLKO MEFs were co-transfected with full-length Bcl-xL and RCaMP for acute rescue experiments. TKO HEK and TKO HeLa cells were transfected with IP3R1/3 constructs for proximity ligation assay. WT, Bcl-xLKO and organelle targeted Bcl-xL expressing stable MEFs were seeded 24 h prior to infection with matrix targeted RCaMP adenovirus at 50, 200 and 75 pfu respectively followed by imaging post 24-48hrs.

#### Fractionation of mef cells

Cells grown overconfluent in T150 flasks (~7 × 10^7^ cells) were harvested using trypsin and washed with Na-Hepes-EGTA (120 mM NaCl, 20mM Na-Hepes, 5mM KCl, 1mM KH_2_PO_4_, 100μM EGTA/Tris pH7.4). All further steps were carried out at 4°C. The cells were subjected to hyposmosis for 10 min (20% ICM: 120mM KCl, 1mM KH_2_PO_4_, 10mM NaCl, 20mM Tris-Hepes and 1μg/mL each of pepstatin, leupeptin and antipain, pH7.4; added to 80% distilled water) supplemented with 5mM MgCl_2_ and 200μM EGTA. Cells were then homogenized on ice using IsoBiotec cell cracker (~11 passes). To restore osmolarity, 3x volume of ICM supplemented with 125mM sucrose, 1μg/mL protease inhibitors and 200μM EGTA pH7.4 were added. To eliminate unbroken cell and nuclei, the cell homogenate was centrifuged at 900g for 10 min. The supernatant was further spun at 2,500g for 10 min for a crude fraction of mitochondria in pellet (M1) followed by centrifugation at 10,000g for 15 min for remaining mitochondrial fraction in pellet (M2), resuspended in 200μL ICM supplemented with 20μM EGTA and protease inhibitors. The supernatant was further centrifuged at 40,000g for 45 min to obtain the ER pellet.

#### Western blot

Reagents for SDS-PAGE were procured from Invitrogen and Bio-Rad. Followed by harvesting, cells were washed twice with ice-cold PBS and solubilized in 1X RIPA lysis buffer (Sigma Aldrich cat# R0278) supplemented with protease inhibitors cocktail (Roche). Lysates were incubated on ice for 20 min followed by centrifugation at 13000xg for 15 min at 4°C. Protein concentrations were estimated with a DC protein assay kit (Bio-Rad). Proteins in cell lysates were resolved on 4–12% SDS-PAGE gels (Invitrogen) and then transferred to nitrocellulose membranes (0.45mm, BioRad). Intercept blocking buffer (LICORbio^™^) was used as blocking reagent. Rabbit monoclonal antibodies against Bcl-xL (1:1000 Cell Signaling #2764), rabbit polyclonal antibodies against IP3R1 (1:5000 custom made, Pocono Rabbit Farm and Laboratories), calnexin (1:1000 Enzo #ADI-SPA-865) and TOM20 (1:1000 Proteintech #11802-1-AP), mouse monoclonal antibodies against cytochrome *c* (1:1000 BD Biosciences #556433), BiP/GRP78 (1:1000 BD Biosciences #610979), Hsp70 (1:1000 Thermofisher #MA3-006) and GAPDH (1:1000 Proteintech #60004-1) were used as primary antibodies. IP3R1 was detected by chemiluminescence using an HRP substrate for CCD imaging (Azure Biosystems). Rabbit HRP-linked secondary antibody (Cell Signaling #7074) was used in the case of IP3R1. For others, corresponding mouse and rabbit LICOR secondary antibodies were used (LICORbio^™^).

#### Cross-linking and co-immunoprecipitation

To determine protein interactions, chemical crosslinking followed by co-immunoprecipitation and western blot were performed. Co-immunoprecipation was performed using Pierce^™^ Classic Magnetic IP/Co-IP kit (Thermofisher #88804). Cell lysates were prepared using the IP lysis buffer provided along with the kit. Chemical crosslinking was induced by treating cell lysates with 200mM bismaleimidohexane (BMH, Thermofisher #22330) for 30 min at room temperature under continuous agitation. The reaction was quenched by treatment with 50mM DTT for 15 min. Crosslinked and control lysates were then incubated with Bcl-xL antibody (1:200) overnight at 4°C under constant agitation. Magnetic beads were added and incubated for 1 h at room temperature under continuous agitation followed by elution according to manufacturer’s protocol (Thermofisher #88804). Elute and corresponding inputs from lysates were subjected to western blot.

#### Intracellular Ca^2+^ measurements

MEFs, HEK and HeLa cells were plated on Poly-D-Lysine coated round (25mm) coverslips which were subjected to loading with 2mM Fura-2-AM (Invitrogen) in a serum-free extracellular medium (ECM) comprising of 121 mM NaCl, 5mM NaHCO_3_, 10mM Na-HEPES, 4.7 mM KCl, 1.2 mM KH_2_PO_4_, 1.2mM MgSO_4_, 2mM CaCl_2_, and 10mM glucose, pH 7.4, containing 2% BSA (Roche) along with 0.003% Pluronic F-127 and 100 mM sulfinpyrazone for 12–15 min at 37°C to record changes in [Ca^2+^]_c_. Following dye loading, cells were washed with fresh ECM with 0.25% BSA and transferred to thermostatic stage (37°C) of the microscope and imaging was continued in 0.25% BSA devoid of Ca^2+^. For intracellular Ca^2+^ imaging the EMCCD camera (Hamamatsu) was fixed to an Olympus IX81 microscope with Lambda DG4 light source controlled by Metamorph (Molecular Devices). The setup used the UV optimized Olympus UAPO/340 x40/1.35 N.A. oil immersion objective. To simultaneously measure [Ca^2+^]_c_ and [Ca^2+^]_m_ Fura-2 fluorescence was recorded at 340 and 380 nm, mitochondrial matrix-targeted RCaMP at 577/20 nm excitations, using dual-band dichroic and emission filters (Chroma, set 73100/59022 respectively). Images were acquired ranging from every 0.1–2 s. Fura-2 was calibrated *in vitro* by adding 1 mM CaCl_2_ followed by 10 mM EGTA/Tris, pH 8.5. [Ca^2+^]_c_ was estimated using the following equation, [Ca^2+^] = K_d_ * (Sf2/Sb2) * (R-R_min_)/(R_max_-R), where K_d_ is the Ca^2+^ dissociation constant (0.224 mM), R is the ratio of fluorescence intensities at 340/380 nm excitation, R_min_ and R_max_ are the fluorescence ratios in Ca^2+^ free and Ca^2+^ saturated conditions, respectively.

#### Photorelease of caged IP_3_

mtRCaMP infected cells were incubated with membrane permeant IP_3_ (10μM, D-2,3-*O*-Isopropylidene-6-*O*-(2-Nitro-4,5-Dimethoxy)benzyl-myo-Inositol 1,4,5 Trisphosphate-Hexakis(Propionoxymethyl) ester, SiChem GmbH #1009832-82-9) for 30 min at 37°C in ECM supplemented with 0.003% Pluronic F-127 and 100mM sulfinpyrazone and 5μM Cal520 (AAT Bioquest #21130) to measure [Ca^2+^]_c_. Cells were then washed with fresh ECM containing 0.25%BSA and transferred to thermostatic stage (37°C) of the microscope where imaging was executed in 0.25% BSA devoid of Ca^2+^. After recording a baseline for 20 s, photo release of caged IP3 was performed with a 365nm UV laser for a pulse of 250–350 msecs. Cal520 and mtRCaMP fluorescence signals were recorded using 480 nm and 577 nm excitations respectively and depicted as F/F_0_. The background signal recorded in cell free regions was employed to correct the fluorescence intensities.

#### Fluorometric Ca^2+^ measurements

[Ca^2+^]_c_ and Ѱ_m_ was measured using a fluorometric assay previously described.^52,53^ Saponin permeabilized MEFs (2*10 ^6) were resuspended in 1.5 mL of intracellular medium (ICM) containing 120 mM KCl, 10 mM NaCl, 1 mM KH_2_PO_4_, 20 mM Tris-HEPES at pH 7.2, augmented with protease inhibitors (leupeptin, antipain, pepstatin at 1μg/mL each), 2mM Mg-ATP, 2μM thapsigargin and maintained in a thermostatic cuvette with continuous stirring at 35°C. Assays were conducted in presence of 20 μM CGP-37157 and 1mM succinate using a multiwavelength-excitation dual-wavelength-emission fluorimeter (DeltaRAM, PTI). The extramitochondrial Ca^2+^ concentration ([Ca^2+^]_c_) was assessed using the ratiometric Ca^2+^ probe Fura-FF (1 mM, AAT Bioquest). Ѱ_m_ was measured with 1.5 mM TMRM (Invitrogen). Fura and TMRM fluorescence were captured simultaneously using 340–380 nm excitation and 500 nm emission, 545 nm excitation and 580 nm emission, respectively. Complete depolarization (maximum de-quench of TMRM fluorescence) happened on application of protonophore FCCP (5 mM). Calibration of the Fura signal was done at the end of each measurement, adding 1 mM CaCl_2_, followed by 10 mM EGTA/Tris, pH 8.5.

#### Proximity ligation assay

Duolink PLA technology incorporated Duolink *In Situ* Red/Green Starter Kit Mouse/Rabbit (MilliporeSigma, cat# DUO92101, DUO92014) was employed to study the interaction of IP3R and Bcl-xL. TKO HEK and TKO HeLa cells, rescued with IP3R1FLAG and IP3R3GFP were seeded on 10mm coverslips. 24–48 h post transfection, cells were fixed in prewarmed 4% PFA for 20 min at RT and washed with PBS (X 3 times). Triton X-100 (0.1%) was then used for 1 min to permeabilize cells, subsequently washed with PBS and incubated with AF488-Phalloidin (in case of IP3R1, diluted in 1% FBS/PBS) for 1 h in dark. HEK cells stably rescued with IP3R2, MEFs expressing wilt-type Bcl-xL and stably expressing organelle specific Bcl-xL along with Bcl-xLKO cells, Bcl-xLKO cells acutely expressing mutant BclxLK87D were stained with MitoBrilliant 646 (Tocris, cat#7700) for 30 min, IP3R2 stable and TKO HEK cells were treated with Alexa Fluor 555 conjugated Wheat Germ Agglutinin (WGA) (Invitrogen, cat# W32464), MEFs with Alexa 488 conjugated WGA (Invitrogen, cat# W11261) for 10mins followed by fixation with chilled 100% methanol for 10 min at −20°C. Cells were then washed with PBS and incubated with Duolink blocking buffer for 1 h followed by incubation with primary antibodies (diluted in Duolink antibody diluent) for 2 h at room temperature. The primary antibodies (1:250) used were anti-FLAG (Sigma Cat# F1804), anti-GFP (Invitrogen #A11120), anti-IP3R2 (1:50, Santa Cruz #398434), anti-IP3R3 (1:50, BD Biosciences #610312) and Bcl-xL (Cell Signaling #2764). Cells were then washed with 5% BSA (15 min X 3) followed by consequent incubation with Duolink secondary antibodies for 1 h at 37°C. After that cells were washed with Duolink wash buffer A (5 min X 3) and incubated with Duolink ligase for 30 min at 37°C. Thereafter cells were washed with Duolink wash buffer A (2 min X 3) and incubated with Duolink polymerase and oligonucleotides for 90 min at 37°C. Finally, cells were washed with Duolink wash buffer B (10 min X 3) and mounted on glass slides using ProLong Glass antifade mountant (Invitrogen, cat# P36980). Images were captured within 24 h.

#### Immunocytochemistry

Primary Antibodies used in PLA studies were validated by immunocytochemistry. HEK and MEF cells on coverslips were fixed with 4% paraformaldehyde (PFA) followed by permeabilization with 0.1% Triton or fixed with 100% chilled methanol. Samples were blocked with 10% FBS for 60 min at room temperature, then incubated with mouse monoclonal primary antibodies against FLAG, IP3R2, IP3R3, GFP and rabbit polyclonal anti-Bcl-xL antibody in a wet chamber for 90 min followed by incubation for 60 min with Alexa Fluor 488, AlexaFuorPlus405 and Alexa Fluor 647 anti-mouse and Alexa Fluor 568 anti-rabbit secondary antibodies (Thermofisher).

#### Alpha fold prediction

AlphaFold2-Multimer predictions of the interaction of the C-terminal tail of IP3R1 and Bcl-xL were performed using the Cosmic^2^ platform.^54^ Mouse IP3R1 (Uniprot P11881; 2717-end) and Bcl-xL without its C-terminal membrane binding domain (Q64373; 1–186) were co-folded. 5 predictions were generated from each of 5 separate models and the overall best ranked prediction is shown in Figure S6A.

### QUANTIFICATION AND STATISTICAL ANALYSIS

Sigma Plot 12.5 and Graphpad Prism 9.0 were used for plotting traces and bar graphs. Unpaired Student’s *t* test, one way-ANOVA were predominantly used for statistical analyses. Experiments were repeated 2–5 times. Details of statistical tests used, along with number of cells, number of experiments, *P*-values, SEM can be found in the figures, figure legends and results sections.

## Supplementary Material

1

Supplemental information can be found online at https://doi.org/10.1016/j.celrep.2026.117767.

## Figures and Tables

**Figure 1.**
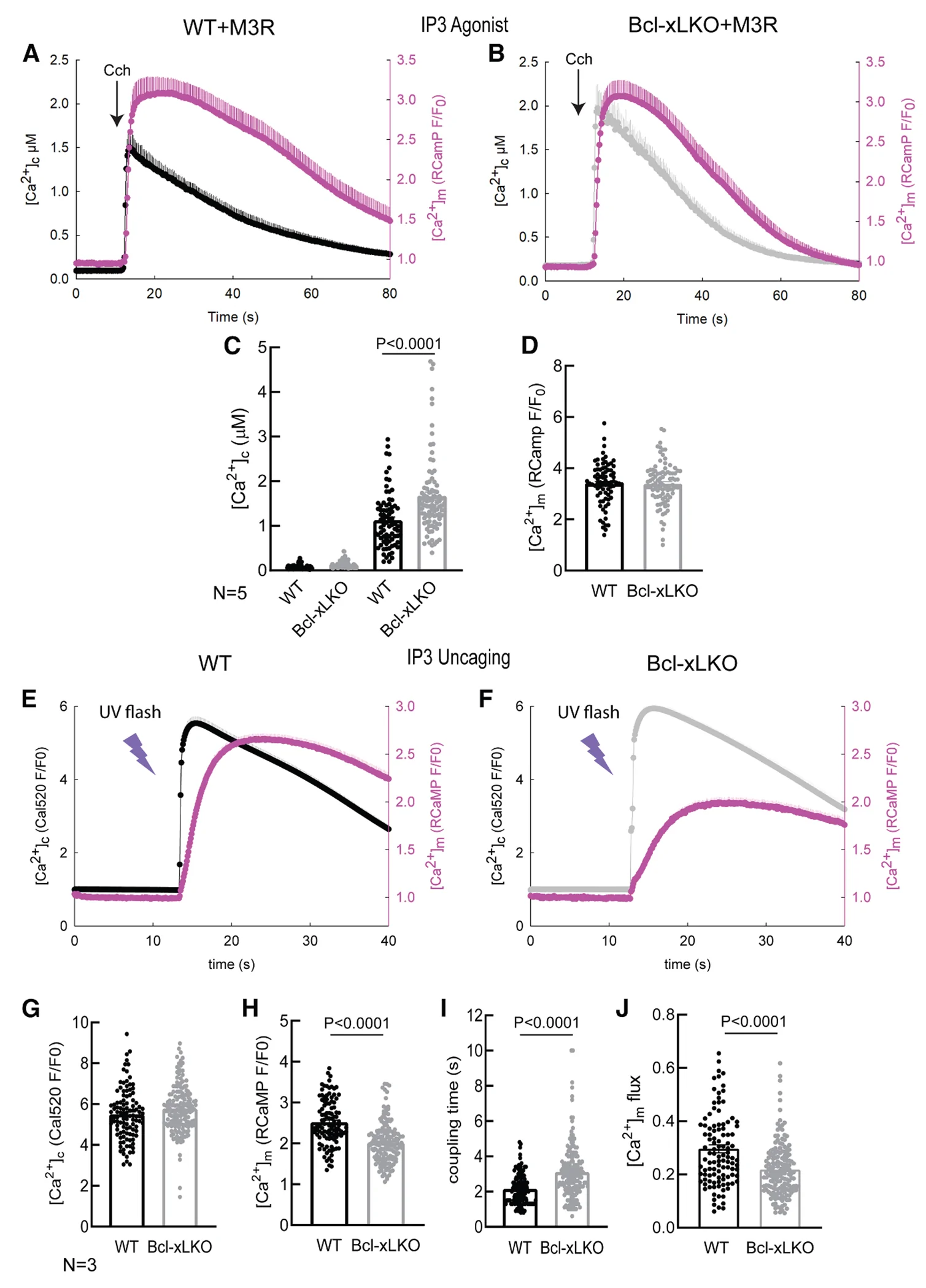
Bcl-xL facilitates IP3R-mediated local ER-mitochondria Ca^2+^ transfer (A) Representative traces of carbachol (100 μM) induced [Ca^2+^]_c_ (black) and corresponding [Ca^2+^]_m_ signals (pink) in WT (*n* = 11) MEF cells expressing M3R. (B) Carbachol induced [Ca^2+^]_c_ (gray) and corresponding [Ca^2+^]_m_ signals (pink) in Bcl-xLKO (*n* = 18) MEF cells expressing M3R. (C) Bar plot shows basal [Ca^2+^]_c_ and carbachol-induced [Ca^2+^]_c_ peaks in WT (black) and Bcl-xLKO (gray) MEFs. Statistical analysis between the groups was determined using one-way ANOVA. (D) Corresponding [Ca^2+^]_m_ peaks in WT and Bcl-xLKO (*n* = 78–92, coming from *N* = 5 experiments). (E) Average traces show [Ca^2+^]_c_ (black) and corresponding [Ca^2+^]_m_ (pink) transients evoked by flash photolysis of caged IP_3_ (10 μM) in WT (*n* = 48) MEF cells. (F) Average traces show simultaneous [Ca^2+^]_c_ (gray) and [Ca^2+^]_m_ (pink) signals upon IP_3_ uncaging in Bcl-xLKO (*n* = 61) MEF cells. (G) IP_3_-triggered [Ca^2+^]_c_ peaks in WT (black) and Bcl-xLKO (gray). (H) Corresponding [Ca^2+^]_m_ peaks in WT and Bcl-xLKO. Statistical analysis between the groups was determined using Students *t* test. (I) Calculated coupling time shows difference in the time to reach half-max [Ca^2+^]_c_ peak and half max [Ca^2+^]_m_ peak indicating ER-mitochondria coupling between WT (black) and Bcl-xLKO (gray). Statistical analysis between the groups was determined using Students *t* test. (J) Instantaneous [Ca^2+^]_m_ rise plotted as [Ca^2+^]_m_ flux in WT (black) and Bcl-xLKO (gray) (*n* = 109–160, from *N* = 3 experiments). Statistical analysis between the groups was determined using Students *t* test. Cell

**Figure 2.**
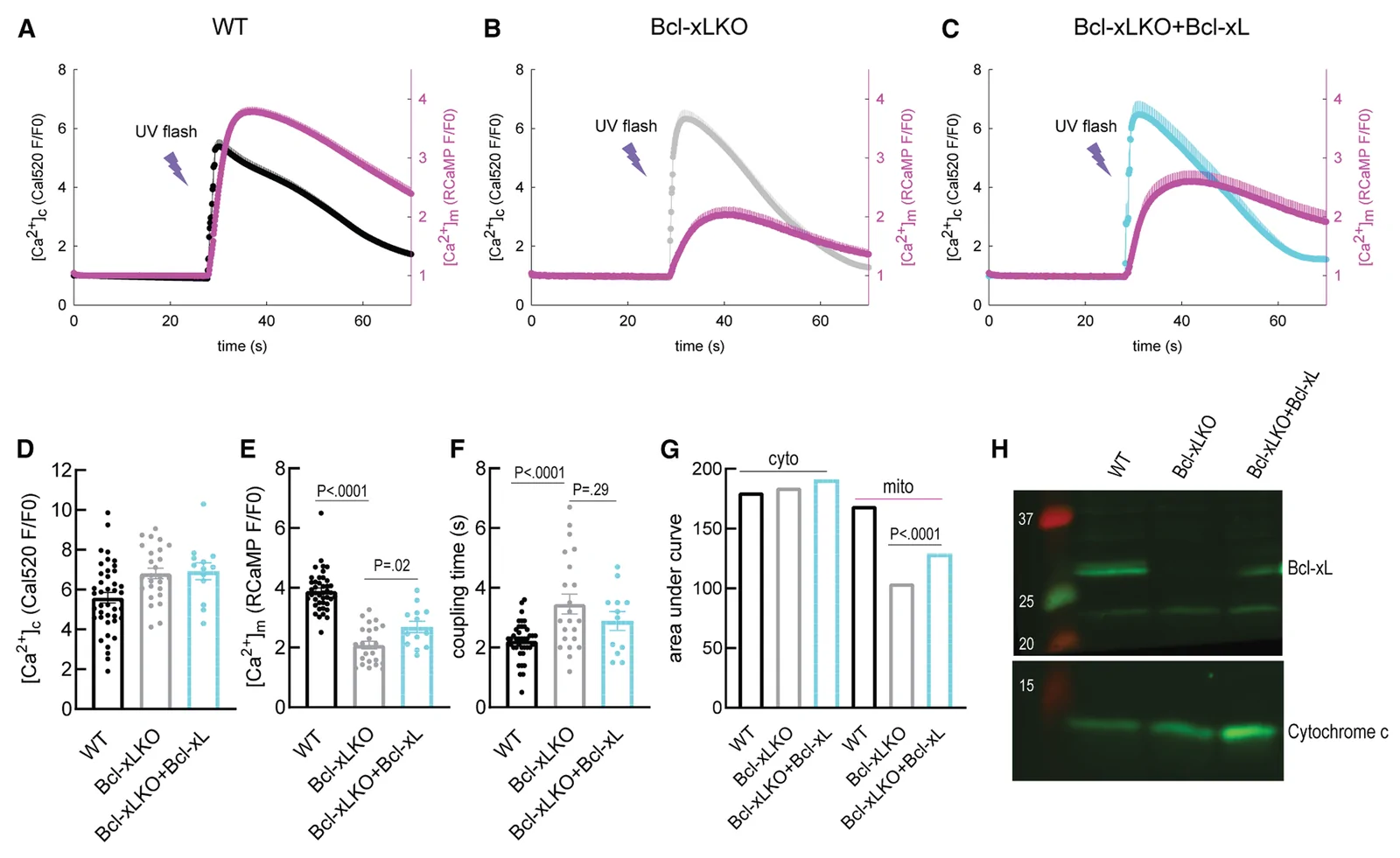
Acute rescue of full-length Bcl-xL restores ER-mitochondria Ca**2+** transfer partially (A) Average traces of IP_3_ (10 μM) induced simultaneous [Ca^2+^]_c_ (black) and [Ca^2+^]_m_ (pink) signals in WT (*n* = 42) MEF cells. (B) Representative traces show IP_3_-induced [Ca^2+^]_c_ (gray) and [Ca^2+^]_m_ (pink) signals in Bcl-xLKO (*n* = 26). (C) Average [Ca^2+^]_c_ (cyan) and [Ca^2+^]_m_ (pink) transients of Bcl-xLKO MEF cells acutely rescued (24–48 h) with full-length Bcl-xL (*n* = 13) in response to IP_3_ uncaging. (D) IP_3_ evoked [Ca^2+^]_c_ peaks in WT (black, *n* = 42), Bcl-xLKO (gray, *n* = 26), and Bcl-xLKO acutely rescued with Bcl-xL (cyan, *n* = 13). (E) Bar plot showing corresponding [Ca^2+^]_m_ peaks in WT, Bcl-xLKO and acute rescue conditions. Statistical analysis between the groups was determined using one-way ANOVA. (F) Coupling time between [Ca^2+^]_c_ and [Ca^2+^]_m_ rises calculated and plotted for WT (*n* = 42), Bcl-xLKO (*n* = 22) and acute rescue (*n* = 12). Statistical analysis between the groups was determined using one-way ANOVA. (G) Bar plot showing area under the curve for cyto and mito measurements calculated from mean traces shown in A, B, C for WT, Bcl-xLKO, and Bcl-xLKO rescued with Bcl-xL, respectively. Statistical analysis between the groups was determined using one-way ANOVA. (H) Western blot showing Bcl-xL (26KDa) specific bands in WT and acute rescue MEF lysates. Cytochrome *c* was used as a loading control.

**Figure 3.**
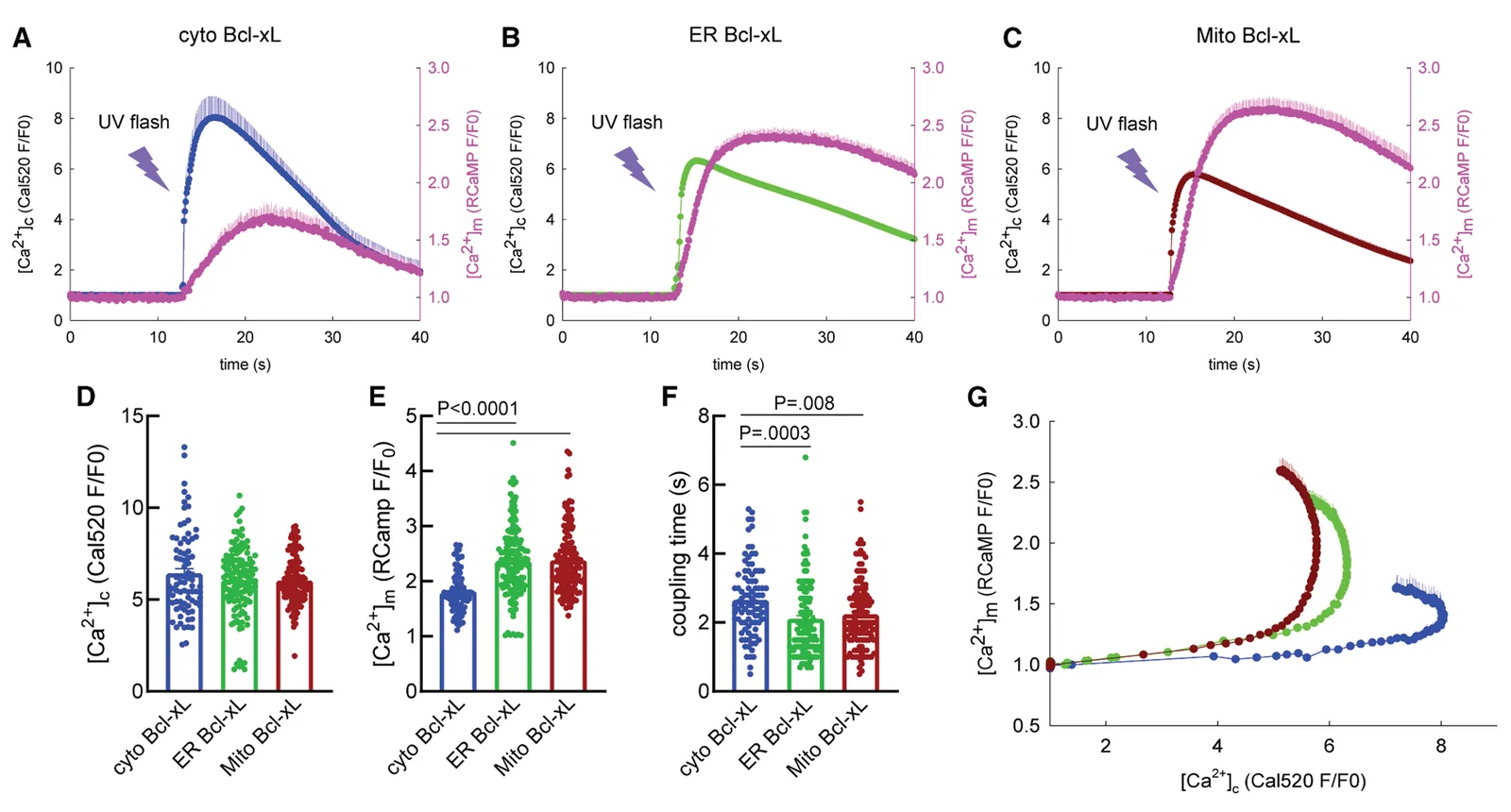
Membrane-targeted Bcl-xL restores ER-mitochondria Ca^2+^ transfer (A) Representative traces of [Ca^2+^]_c_ (blue) and corresponding [Ca^2+^]_m_ (pink) transients evoked by flash photolysis of caged IP_3_ (10 μM) in stable MEF cells expressing cytoplasm-targeted Bcl-xL (*n* = 11). (B) Average traces showing IP_3_-induced [Ca^2+^]_c_ (green) and corresponding [Ca^2+^]_m_ (pink) signals in stable MEF cells expressing ER-targeted Bcl-xL (*n* = 55). (C) Average traces of [Ca^2+^]_c_ (burgundy) and subsequent [Ca^2+^]_m_ (pink) transients in response to IP_3_ uncaging in MEF cells expressing mitochondria-localized Bcl-xL (*n* = 47). (D) IP_3_-induced [Ca^2+^]_c_ peaks in the case of cyto Bcl-xL (blue), ER Bcl-xL (green) and Mito Bcl-xL (burgundy). (E) Corresponding [Ca^2+^]_m_ peaks in cyto Bcl-xL (*n* = 92), ER Bcl-xL (*n* = 140), and Mito Bcl-xL (*n* = 144). Statistical analysis between the groups was determined using one-way ANOVA. (F) Bar plots showing calculated coupling time for MEF cells expressing cyto Bcl-xL (*n* = 92), ER Bcl-xL (*n* = 136), and Mito Bcl-xL (*n* = 141) coming from *N* = 5 experiments. Statistical analysis between the groups was determined using one-way ANOVA. (G) Mean traces showing IP_3_-induced [Ca^2+^]_m_ signals plotted as a function of [Ca^2+^]_c_ signals for stable MEF cells expressing Mito Bcl-xL, ER Bcl-xL and cyto Bcl-xL.

**Figure 4.**
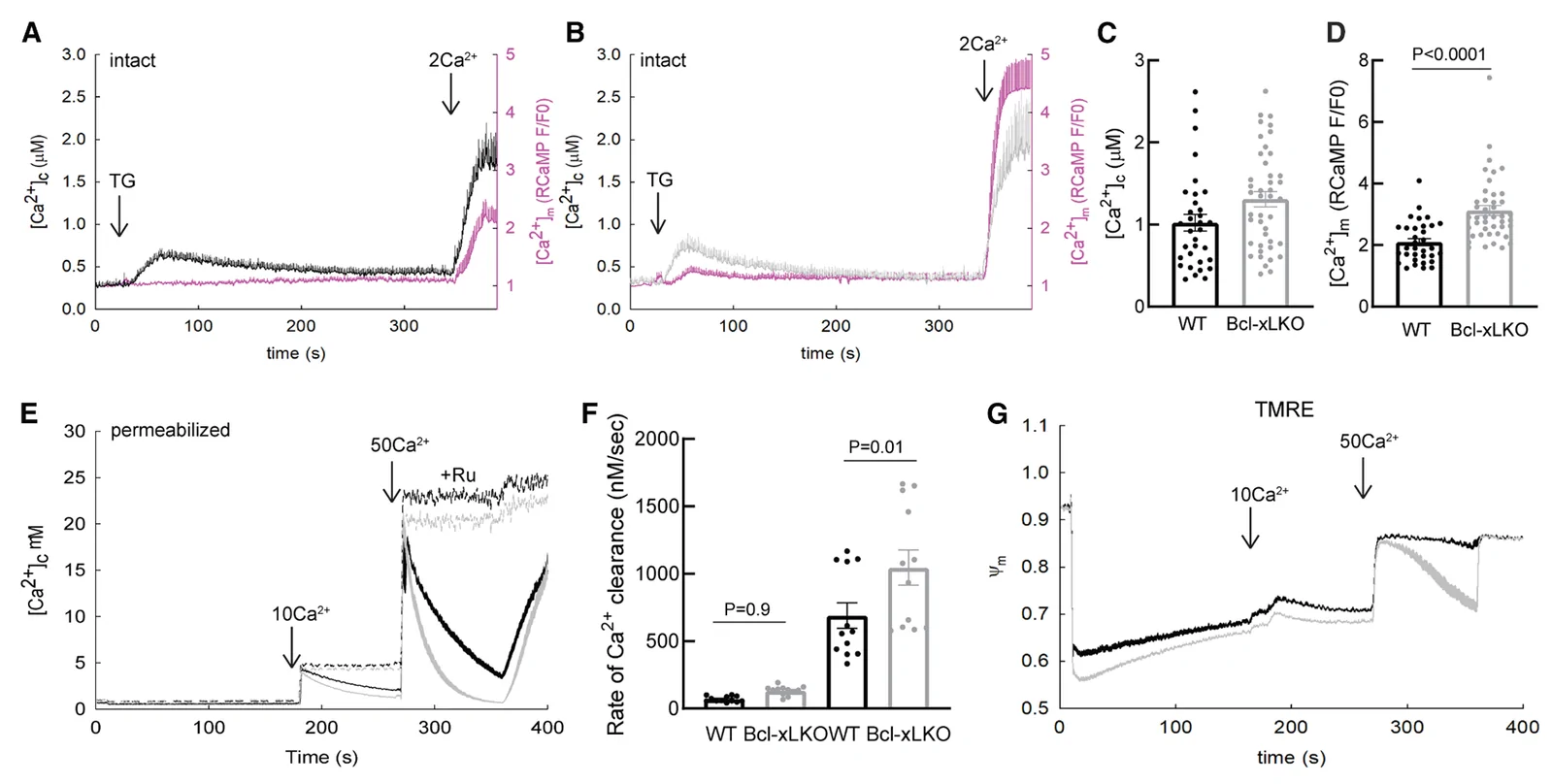
Bcl-xL is dispensable for mitochondrial Ca^2+^ uptake upon global Ca^2+^ rise (A) Average traces of [Ca^2+^]_c_ (black) and [Ca^2+^]_m_ (pink) signals showing SOCE triggered by thapsigargin (2 μM) induced ER Ca^2+^ depletion in WT (*n* = 10). (B) Same in Bcl-xLKO (*n* = 10). (C) SOCE-derived [Ca^2+^]_c_ peaks in WT (black), Bcl-xLKO (gray). (D) Corresponding SOCE-derived [Ca^2+^]_m_ signals in WT (*n* = 33), Bcl-xLKO (*n* = 43). Statistical analysis between the groups was determined using Students *t* test. (E) Mean traces (*n* = 3) show Ru265 (1 μM) sensitive extramitochondrial Ca^2+^ measurement in response to low Ca^2+^ (10 mM) and high Ca^2+^ (50 mM) boluses in WT (black) and Bcl-xLKO (gray). (F) Bar plot shows rates of extramitochondrial clearance for low and high Ca^2+^ doses in WT and Bcl-xLKO for *n* = 12 runs coming from *N* = 4 experiments. Statistical analysis between the groups was determined using one-way ANOVA. (G) Average traces show mitochondrial membrane potential in WT and Bcl-xLKO MEF cells during low and high Ca^2+^ addition.

**Figure 5.**
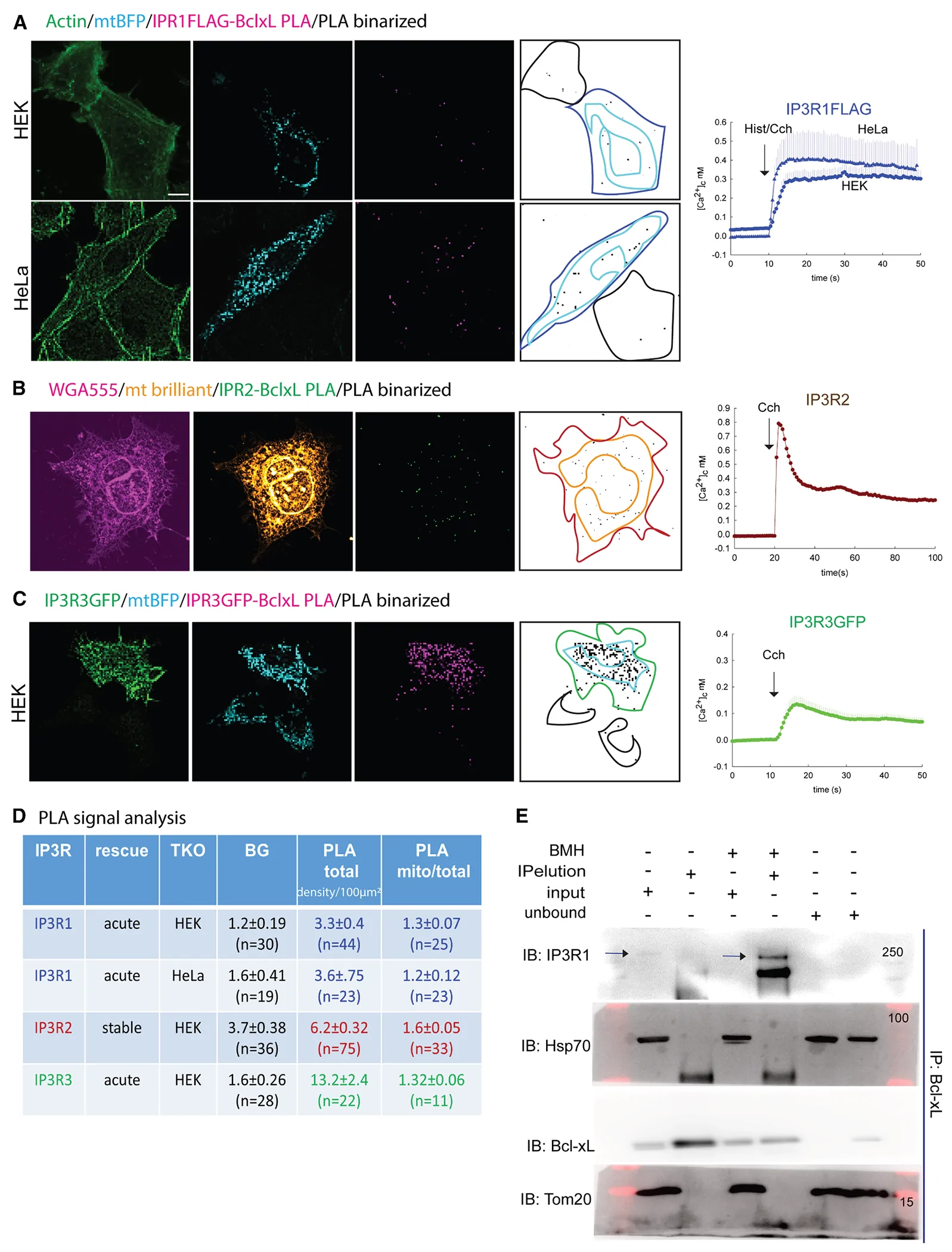
Interaction of Bcl-xL and IP3R in the context of mitochondrial localization (A) Representative fixed (PFA) cell images from proximity ligation assay (PLA) conducted between IP3R1 and Bcl-xL to assess interaction in TKO HEK and TKO HeLa cells rescued with IP3R1FLAG. mtBFP was used as a mitochondrial marker. Cells were stained with phalloidin 488 to label actin filaments to identify cell area prior to PLA staining. Bar graphs showing density of PLA dots (no. per 100 μm^2^) calculated in the entire cell and mitochondrial regions in TKO HEK and TKO HeLa rescued with IP3R1FLAG and untransfected control. In the same batch of cells transfected with IP3R1FLAG, functions of IP3R1 were validated by agonist (carbachol/histamine) induced Ca^2+^ measurements in HEK and HeLa cells (n~10). (B) Representative fixed (methanol) images from PLA conducted between IP3R2 and Bcl-xL in TKO HEK stably rescued with IP3R2. These cells were stained with WGA555 and Mito Brilliant 646 to track the cell area and mitochondria (orange), respectively. TKO HEK cells were used as a control. The function of IP3R2 was simultaneously validated with carbachol-induced Ca^2+^ transients in the stable cells (n~30). (C) Representative fixed (PFA) cell images from PLA executed between IP3R3 and Bcl-xL to assess interaction in TKO HEK cells acutely rescued with IP3R3GFP. mtBFP was co-transfected to serve as the mitochondrial marker. Function of IP3R3 was checked with carbachol stimulation of the same batch of cells (n~15). (D) PLA signal was quantified as density of PLA dots (no. per 100 μm^2^) calculated in entire cell (total) and mitochondrial regions (mito). The table describes the PLA signal obtained in total cell vs. mitochondria using the IP3R isoforms in different cells in comparison to control (BG). (E) Interaction of IP3R1 and Bcl-xL was also assessed in crosslinking (+BMH) followed by immunoprecipitation (using anti-Bcl-xL antibody) in Mito Bcl-xL expressing stable cells (MEFs). IP3R1 bands marked in input and crosslinked IP elute. Grp78 and Tom20 were used as ER- and mitochondria-localized proteins, respectively.

**Figure 6.**
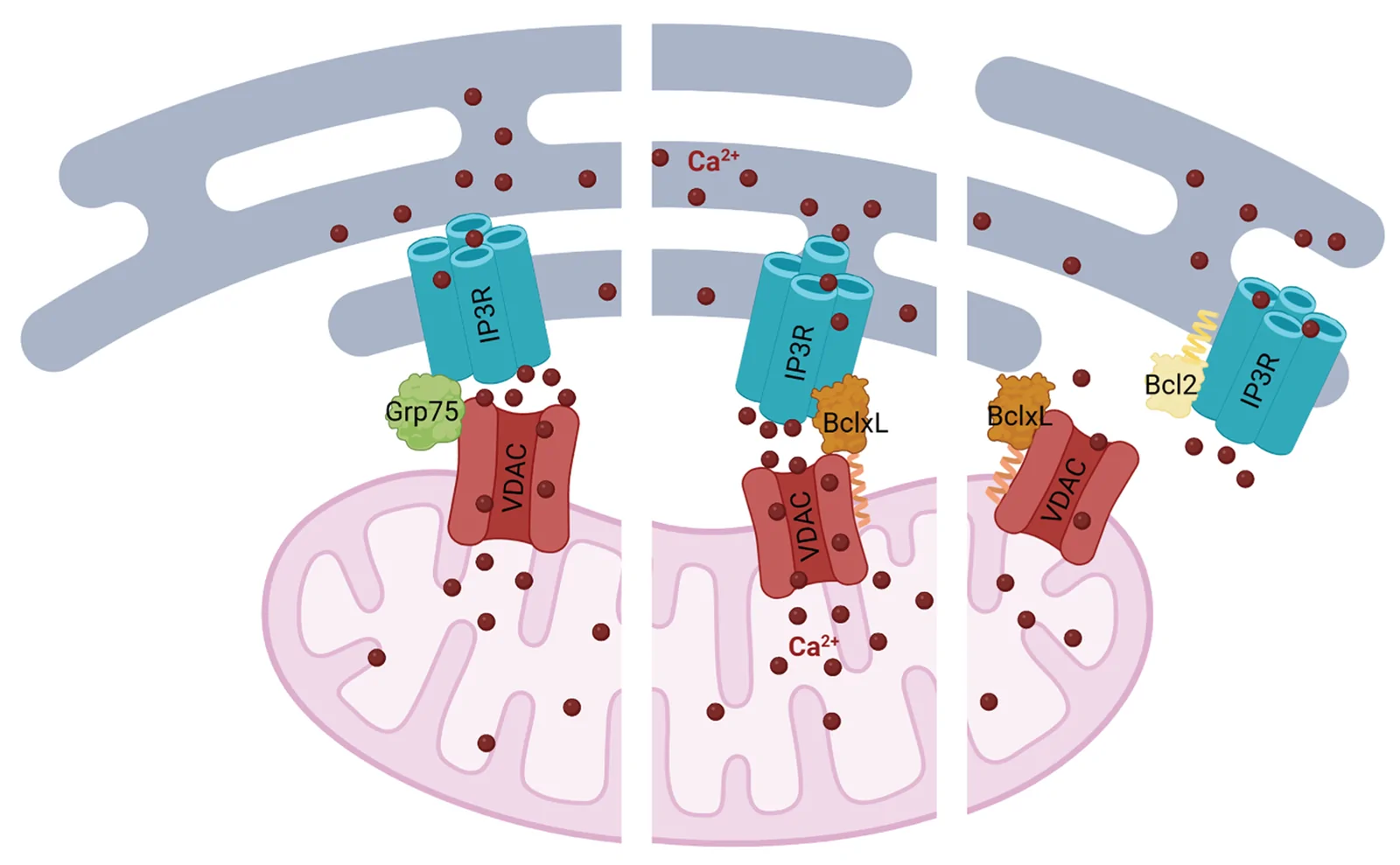
Speculative scheme representing plausible IP3R-Bcl-xL-VDAC tethering complex The left panel of the scheme shows the already proposed IP3R-Grp75-VDAC tripartite complex. The right panel shows lateral interaction of Bcl-xL/Bcl2 with VDAC/IP3R. In the middle is a proposition of an IP3R-Bcl-xL-VDAC trans interacting complex.

**Table:** KEY RESOURCES TABLE

REAGENT or RESOURCE	SOURCE	IDENTIFIER
Antibodies		
Rabbit anti-Bcl-xL	Cell Signaling Technology	2764 RRID: AB_2228008
Rabbit anti-IP3R1	Pocono Rabbit Farm and Laboratories	N/A
Rabbit anti-calnexin	Enzo Life Sciences	ADI-SPA-865 RRID: AB_10618434
Rabbit anti-TOM20	Proteintech	11802-1-AP RRID: AB_2207530
Mouse anti-cytochrome C	BD Biosciences	556433 RRID: AB_396417
Mouse anti-BiP GRP78	BD Biosciences	610979 RRID: AB_398292
Mouse anti-HSP70	Thermo Fisher Scientific	MA3-006 RRID: AB_325454
Mouse anti-GAPDH	Proteintech	60004–1 RRID: AB_2107436
Mouse anti-IP3R2	Santa Cruz	398434 RRID: AB_2924300
Mouse anti-IP3R3	BD Biosciences	610312 RRID: AB_397705
Mouse anti-FLAG	Sigma-Aldrich	F1804 RRID: AB_262044
Mouse anti-GFP	Thermo Fisher Scientific	A11120 RRID:AB_221568
HRP-linked goat anti-rabbit IgG	Cell Signaling Technology	7074 RRID: AB_2099233
IR Dye goat anti-rabbit 800CW	LI-COR	926–32211 RRID: AB_621843
IR Dye goat anti-mouse 800CW	LI-COR	926–32210 RRID: AB_621842
Alexa Fluor^™^ 488	Thermofisher	A-21200 RRID: AB_2535786
Alexa Fluor^™^ 568	Thermofisher	A-11036 RRID: AB_10563566
Alexa Fluor^™^ 647	Thermofisher	A-21463 RRID: AB_2535869
Alexa Fluor^™^ Plus 405	Thermofisher	A48255 RRID: AB_2890536
Virus strain		
Ad-2mt-RcaMP1 (amp) Backbone-Adenoviral-Human Type 5 (dE1/E3)	VECTOR BIOLABS	20200324T#5
Chemicals, peptides, and recombinant proteins		
Fura-2, AM, cell permeant	ThermoFisher	F1221
Cal-520^®^, AM	AAT Bioquest	21130
Caged IP_3_	SiChem	cag-iso-2-145-100
Fura-FF salt	AATBioquest	21028
Fura2 salt	TefLabs	0–104
Tetramethylrhodamine, Methyl Ester, Perchlorate (TMRM)	ThermoFisher	T668
Thapsigargin	Enzo Life Sciences	BML-PE180
CGP-37157	Enzo Life Sciences	BML-CM119
HBSS	Gibco	24020117
DMEM	Gibco	11965118
Glutamine	Gibco	A2916801
Penicillin-Streptomycin (10,000 U/mL)	Gibco	15140122
Trypsin-EDTA	Gibco	25200056
RIPA buffer	Sigma Aldrich	RO278
Leupeptin	Sigma Aldrich	L2884
Antipain	Sigma Aldrich	A6191
Pepstatin	Sigma Aldrich	P5318
TBST	Millipore Sigma	T9039
PFA	Electron Microscopy Sciences	15714S
BMH (bismaleimidohexane)	Thermofisher	22330
poly-D-lysine	Gibco	A3890401
FCCP	Sigma	C2920
Oligomycin	Sigma	O4876
MitoBrilliant^™^ 646	Tocris	7700
Alexa Fluor 555 conjugated Wheat Germ Agglutinin (WGA)	Thermofisher	W32464
Alexa 488 conjugated WGA	Thermofisher	W11261
ProLong^™^ Glass Antifade Mountant	Thermofisher	P36980
NuPAGE^™^ MOPS SDS Buffer Kit	Invitrogen	NP0050
Critical commercial assays		
DC Protein Assay	Biorad	5000112
Lipofectamine 3000	Invitrogen	L3000015
Pierce^™^ Classic Magnetic IP/Co-IP Kit	Thermofisher	88804
Duolink^®^ *In Situ* Red Starter Kit Mouse/Rabbit	Sigma-Aldrich	DUO92101
Duolink^®^ *In Situ* Detection Reagents Green	Sigma-Aldrich	DUO92014
Experimental models: Cell lines (Cell line authentication have not been performed; periodically checked and tested negative for Mycoplasma contamination)		
WT and Bcl-xLKO MEFs	Eno et al.^31^	N/A
Cyto Bcl-xL(Δc), ER Bcl-xL (cb5), Mito Bcl-xL (ActA) stably rescued MEFs	Eno et al.^31^	N/A
IP3R1/2/3 triple knockout (TKO) HEK and HeLa	Bartok et al.^8^	N/A
Recombinant DNA		
mtRCaMP	Bartok et al.^8^	N/A
Bcl-xL and Bcl-xLK87D	Rosa et al.^26^	N/A
IP3R1FLAG, IP3R3GFP	Bartok et al.^8^	N/A
Software and algorithms		
GraphPad Prism 9.3.0.	GraphPad	N/A
SigmaPlot 12.5	Systat Software	N/A
Fiji 2.14.0	NIH	N/A
Illustrator	Adobe Inc.	N/A
Biorender	Science Suit Inc.	N/A
MS Excel	Microsoft	N/A

## Data Availability

All data reported in this paper will be shared by the lead contact upon request. This paper does not report original code. Any additional information required to reanalyze the data reported in this paper will be made available by the lead contact upon request.

## Highlights

- Bcl-xL in the outer mitochondrial membrane binds to ER IP_3_ receptors in a trans configuration
- This interaction facilitates local calcium transfer at ER-mitochondria contacts
- Bcl-xL is dispensable for mitochondrial calcium uptake triggered by changes in global calcium

## References

[1] Giorgi C, Marchi S, and Pinton P. (2018). The machineries, regulation and cellular functions of mitochondrial calcium. Nat. Rev. Mol. Cell Biol. 19, 713–730. 10.1038/s41580-018-0052-8. 30143745

[2] Rizzuto R, De Stefani D, Raffaello A, and Mammucari C. (2012). Mitochondria as sensors and regulators of calcium signalling. Nat. Rev. Mol. Cell Biol. 13, 566–578. 10.1038/nrm3412. 22850819

[3] Csordás G, Weaver D, and Hajnóczky G. (2018). Endoplasmic Reticulum-Mitochondrial Contactology: Structure and Signaling Functions. Trends Cell Biol. 28, 523–540. 10.1016/j.tcb.2018.02.009. PMC6005738 29588129

[4] Voeltz GK, Sawyer EM, Hajnóczky G, and Prinz WA. (2024). Making the connection: How membrane contact sites have changed our view of organelle biology. Cell 187, 257–270. 10.1016/j.cell.2023.11.040. PMC11830234 38242082

[5] Makio T, and Simmen T. (2024). Not So Rare: Diseases Based on Mutant Proteins Controlling Endoplasmic Reticulum-Mitochondria Contact (MERC) Tethering. Contact. 7, 25152564241261228. 10.1177/25152564241261228. PMC11273598 39070058

[6] Paillusson S, Gomez-Suaga P, Stoica R, Little D, Gissen P, Devine MJ, Noble W, Hanger DP, and Miller CCJ. (2017). alpha-Synuclein binds to the ER-mitochondria tethering protein VAPB to disrupt Ca^2+^ homeostasis and mitochondrial ATP production. Acta Neuropathol. 134, 129–149. 10.1007/s00401-017-1704-z. PMC5486644 28337542

[7] Sassano ML, van Vliet AR, and Agostinis P. (2017). Mitochondria-Associated Membranes As Networking Platforms and Regulators of Cancer Cell Fate. Front. Oncol. 7, 174. 10.3389/fonc.2017.00174. PMC5563315 28868254

[8] Bartok A, Weaver D, Golenár T, Nichtova Z, Katona M, Bánsághi S, Alzayady KJ, Thomas VK, Ando H, Mikoshiba K, (2019). IP_3_ receptor isoforms differently regulate ER-mitochondrial contacts and local calcium transfer. Nat. Commun. 10, 3726. 10.1038/s41467-019-11646-3. PMC6700175 31427578

[9] Szabadkai G, Bianchi K, Várnai P, De Stefani D, Wieckowski MR, Cavagna D, Nagy AI, Balla T, and Rizzuto R. (2006). Chaperone-mediated coupling of endoplasmic reticulum and mitochondrial Ca^2+^ channels. J. Cell Biol. 175, 901–911. 10.1083/jcb.200608073. PMC2064700 17178908

[10] De Stefani D, Rizzuto R, and Pozzan T. (2016). Enjoy the Trip: Calcium in Mitochondria Back and Forth. Annu. Rev. Biochem. 85, 161–192. 10.1146/annurev-biochem-060614-034216. 27145841

[11] Kamer KJ, and Mootha VK. (2015). The molecular era of the mitochondrial calcium uniporter. Nat. Rev. Mol. Cell Biol. 16, 545–553. 10.1038/nrm4039. 26285678

[12] Ivanova H, Vervliet T, Monaco G, Terry LE, Rosa N, Baker MR, Parys JB, Serysheva II, Yule DI, and Bultynck G. (2020). Bcl-2-Protein Family as Modulators of IP(3) Receptors and Other Organellar Ca^2+^ Channels. Cold Spring Harb. Perspect. Biol. 12, a035089. 10.1101/cshperspect.a035089. PMC7111250 31501195

[13] Eckenrode EF, Yang J, Velmurugan GV, Foskett JK, and White C. (2010). Apoptosis protection by Mcl-1 and Bcl-2 modulation of inositol 1,4,5-trisphosphate receptor-dependent Ca^2+^ signaling. J. Biol. Chem. 285, 13678–13684. 10.1074/jbc.M109.096040. PMC2859530 20189983

[14] Monaco G, Beckers M, Ivanova H, Missiaen L, Parys JB, De Smedt H, and Bultynck G. (2012). Profiling of the Bcl-2/Bcl-X(L)-binding sites on type 1 IP(3) receptor. Biochem. Biophys. Res. Commun. 428, 31–35. 10.1016/j.bbrc.2012.10.002. 23058917

[15] Schulman JJ, Wright FA, Kaufmann T, and Wojcikiewicz RJH. (2013). The Bcl-2 protein family member Bok binds to the coupling domain of inositol 1,4,5-trisphosphate receptors and protects them from proteolytic cleavage. J. Biol. Chem. 288, 25340–25349. 10.1074/jbc.M113.496570. PMC3757198 23884412

[16] Distelhorst CW. (2018). Targeting Bcl-2-IP(3) receptor interaction to treat cancer: A novel approach inspired by nearly a century treating cancer with adrenal corticosteroid hormones. Biochim. Biophys. Acta. Mol. Cell Res. 1865, 1795–1804. 10.1016/j.bbamcr.2018.07.020. PMC6345611 30053503

[17] Kerkhofs M, Bittremieux M, Morciano G, Giorgi C, Pinton P, Parys JB, and Bultynck G. (2018). Emerging molecular mechanisms in chemotherapy: Ca^2+^ signaling at the mitochondria-associated endoplasmic reticulum membranes. Cell Death Dis. 9, 334. 10.1038/s41419-017-0179-0. PMC5832420 29491433

[18] White C. (2017). The Regulation of Tumor Cell Invasion and Metastasis by Endoplasmic Reticulum-to-Mitochondrial Ca^2+^ Transfer. Front. Oncol. 7, 171. 10.3389/fonc.2017.00171. PMC5554129 28848710

[19] Ivanova H, Wagner LE 2nd, Tanimura A, Vandermarliere E, Luyten T, Welkenhuyzen K, Alzayady KJ, Wang L, Hamada K, Mikoshiba K, (2019). Bcl-2 and IP(3) compete for the ligand-binding domain of IP(3)Rs modulating Ca^2+^ signaling output. Cell. Mol. Life Sci. 76, 3843–3859. 10.1007/s00018-019-03091-8. PMC11105292 30989245

[20] Rong YP, Aromolaran AS, Bultynck G, Zhong F, Li X, McColl K, Matsuyama S, Herlitze S, Roderick HL, Bootman MD, (2008). Targeting Bcl-2-IP3 receptor interaction to reverse Bcl-2’s inhibition of apoptotic calcium signals. Mol. Cell 31, 255–265. 10.1016/j.molcel.2008.06.014. PMC3660092 18657507

[21] Oakes SA, Scorrano L, Opferman JT, Bassik MC, Nishino M, Pozzan T, and Korsmeyer SJ. (2005). Proapoptotic BAX and BAK regulate the type 1 inositol trisphosphate receptor and calcium leak from the endoplasmic reticulum. Proc. Natl. Acad. Sci. USA 102, 105–110. 10.1073/pnas.0408352102. PMC544078 15613488

[22] Pinton P, Ferrari D, Magalhães P, Schulze-Osthoff K, Di Virgilio F, Pozzan T, and Rizzuto R. (2000). Reduced loading of intracellular Ca^2+^ stores and downregulation of capacitative Ca^2+^ influx in Bcl-2-overex-pressing cells. J. Cell Biol. 148, 857–862. 10.1083/jcb.148.5.857. PMC2174537 10704437

[23] Carpio MA, Means RE, Brill AL, Sainz A, Ehrlich BE, and Katz SG. (2021). BOK controls apoptosis by Ca^2+^ transfer through ER-mitochondrial contact sites. Cell Rep. 34, 108827. 10.1016/j.celrep.2021.108827. PMC7995216 33691099

[24] Tang SX, Camara CM, Franco JA, Pazyra-Murphy MF, Li Y, Godes M, Moyer BM, Bird GH, Segal RA, and Walensky LD. (2024). Dissecting the neuroprotective interaction between the BH4 domain of BCL-w and the IP3 receptor. Cell Chem. Biol. 31, 1815–1826.e5. 10.1016/j.chembiol.2024.06.016. PMC11490406 39067448

[25] Kaufmann T, Schlipf S, Sanz J, Neubert K, Stein R, and Borner C. (2003). Characterization of the signal that directs Bcl-x(L), but not Bcl-2, to the mitochondrial outer membrane. J. Cell Biol. 160, 53–64. 10.1083/jcb.200210084. PMC2172731 12515824

[26] Rosa N, Speelman-Rooms F, Parys JB, and Bultynck G. (2022). Modulation of Ca^2+^ signaling by antiapoptotic Bcl-2 versus Bcl-xL: From molecular mechanisms to relevance for cancer cell survival. Biochim. Biophys. Acta. Rev. Cancer 1877, 188791. 10.1016/j.bbcan.2022.188791. 36162541

[27] White C, Li C, Yang J, Petrenko NB, Madesh M, Thompson CB, and Foskett JK. (2005). The endoplasmic reticulum gateway to apoptosis by Bcl-X(L) modulation of the InsP3R. Nat. Cell Biol. 7, 1021–1028. 10.1038/ncb1302. PMC2893337 16179951

[28] Yang J, Vais H, Gu W, and Foskett JK. (2016). Biphasic regulation of InsP3 receptor gating by dual Ca^2+^ release channel BH3-like domains mediates Bcl-xL control of cell viability. Proc. Natl. Acad. Sci. USA 113, E1953–E1962. 10.1073/pnas.1517935113. PMC4822637 26976600

[29] Monaco G, Decrock E, Arbel N, van Vliet AR, La Rovere RM, De Smedt H, Parys JB, Agostinis P, Leybaert L, Shoshan-Barmatz V, and Bultynck G. (2015). The BH4 domain of anti-apoptotic Bcl-XL, but not that of the related Bcl-2, limits the voltage-dependent anion channel 1 (VDAC1)-mediated transfer of pro-apoptotic Ca^2+^ signals to mitochondria. J. Biol. Chem. 290, 9150–9161. 10.1074/jbc.M114.622514. PMC4423701 25681439

[30] Huang H, Hu X, Eno CO, Zhao G, Li C, and White C. (2013). An interaction between Bcl-xL and the voltage-dependent anion channel (VDAC) promotes mitochondrial Ca^2+^ uptake. J. Biol. Chem. 288, 19870–19881. 10.1074/jbc.M112.448290. PMC3707689 23720737

[31] Eno CO, Eckenrode EF, Olberding KE, Zhao G, White C, and Li C. (2012). Distinct roles of mitochondria- and ER-localized Bcl-xL in apoptosis resistance and Ca^2+^ homeostasis. Mol. Biol. Cell 23, 2605–2618. 10.1091/mbc.E12-02-0090. PMC3386223 22573883

[32] Gadet R, Jabbour L, Nguyen TTM, Lohez O, Mikaelian I, Gonzalo P, Luyten T, Chalabi-Dchar M, Wierinckx A, Marcillat O, (2024). The endoplasmic reticulum pool of Bcl-xL prevents cell death through IP3R-dependent calcium release. Cell Death Discov. 10, 346. 10.1038/s41420-024-02112-1. PMC11294475 39090104

[33] Pfeiffer A, Schneider J, Bueno D, Dolga A, Voss TD, Lewerenz J, Wüllner V, and Methner A. (2017). Bcl-x(L) knockout attenuates mitochondrial respiration and causes oxidative stress that is compensated by pentose phosphate pathway activity. Free Radic. Biol. Med. 112, 350–359. 10.1016/j.freeradbiomed.2017.08.007. 28807815

[34] Lalier L, Mignard V, Joalland MP, Lanoé D, Cartron PF, Manon S, and Vallette FM. (2021). TOM20-mediated transfer of Bcl2 from ER to MAM and mitochondria upon induction of apoptosis. Cell Death Dis. 12, 182. 10.1038/s41419-021-03471-8. PMC7884705 33589622

[35] Wu S, Lu Q, Wang Q, Ding Y, Ma Z, Mao X, Huang K, Xie Z, and Zou MH. (2017). Binding of FUN14 Domain Containing 1 With Inositol 1,4,5-Trisphosphate Receptor in Mitochondria-Associated Endoplasmic Reticulum Membranes Maintains Mitochondrial Dynamics and Function in Hearts in Vivo. Circulation 136, 2248–2266. 10.1161/CIRCULATIONAHA.117.030235. PMC5716911 28942427

[36] Filadi R, Leal NS, Schreiner B, Rossi A, Dentoni G, Pinho CM, Wiehager B, Cieri D, Calí T, Pizzo P, and Ankarcrona M. (2018). TOM70 Sustains Cell Bioenergetics by Promoting IP3R3-Mediated ER to Mitochondria Ca^2+^ Transfer. Curr. Biol. 28, 369–382.e6. 10.1016/j.cub.2017.12.047. 29395920

[37] Carreras-Sureda A, Jaña F, Urra H, Durand S, Mortenson DE, Sagredo A, Bustos G, Hazari Y, Ramos-Fernández E, Sassano ML, (2019). Non-canonical function of IRE1alpha determines mitochondria-associated endoplasmic reticulum composition to control calcium transfer and bioenergetics. Nat. Cell Biol. 21, 755–767. 10.1038/s41556-019-0329-y. PMC7246037 31110288

[38] Li C, Wang X, Vais H, Thompson CB, Foskett JK, and White C. (2007). Apoptosis regulation by Bcl-x(L) modulation of mammalian inositol 1,4,5-trisphosphate receptor channel isoform gating. Proc. Natl. Acad. Sci. USA 104, 12565–12570. 10.1073/pnas.0702489104. PMC1941509 17636122

[39] Rosa N, Ivanova H, Wagner LE 2nd, Kale J, La Rovere R, Welkenhuyzen K, Louros N, Karamanou S, Shabardina V, Lemmens I, (2022). Bcl-xL acts as an inhibitor of IP(3)R channels, thereby antagonizing Ca^2+^-driven apoptosis. Cell Death Differ. 29, 788–805. 10.1038/s41418-021-00894-w. PMC8990011 34750538

[40] Csordás G, Thomas AP, and Hajnóczky G. (1999). Quasi-synaptic calcium signal transmission between endoplasmic reticulum and mitochondria. EMBO J. 18, 96–108. 10.1093/emboj/18.1.96. PMC1171106 9878054

[41] Hayashi T, and Su TP. (2007). Sigma-1 receptor chaperones at the ER-mitochondrion interface regulate Ca^2+^ signaling and cell survival. Cell 131, 596–610. 10.1016/j.cell.2007.08.036. 17981125

[42] Liu Y, Ma X, Fujioka H, Liu J, Chen S, and Zhu X. (2019). DJ-1 regulates the integrity and function of ER-mitochondria association through interaction with IP3R3-Grp75-VDAC1. Proc. Natl. Acad. Sci. USA 116, 25322–25328. 10.1073/pnas.1906565116. PMC6911199 31767755

[43] D’Eletto M, Rossin F, Occhigrossi L, Farrace MG, Faccenda D, Desai R, Marchi S, Refolo G, Falasca L, Antonioli M, (2018). Transglutaminase Type 2 Regulates ER-Mitochondria Contact Sites by Interacting with GRP75. Cell Rep. 25, 3573–3581.e4. 10.1016/j.celrep.2018.11.094. 30590033

[44] Erustes AG, D’Eletto M, Guarache GC, Ureshino RP, Bincoletto C, da Silva Pereira GJ, Piacentini M, and Smaili SS. (2021). Overexpression of alpha-synuclein inhibits mitochondrial Ca^2+^ trafficking between the endoplasmic reticulum and mitochondria through MAMs by altering the GRP75-IP3R interaction. J. Neurosci. Res. 99, 2932–2947. 10.1002/jnr.24952. 34510532

[45] McNally MA, Soane L, Roelofs BA, Hartman AL, and Hardwick JM. (2013). The N-terminal helix of Bcl-xL targets mitochondria. Mitochondrion 13, 119–124. 10.1016/j.mito.2013.01.004. PMC3650083 23333404

[46] Arbel N, Ben-Hail D, and Shoshan-Barmatz V. (2012). Mediation of the antiapoptotic activity of Bcl-xL protein upon interaction with VDAC1 protein. J. Biol. Chem. 287, 23152–23161. 10.1074/jbc.M112.345918. PMC3391160 22589539

[47] Baker MR, Fan G, and Serysheva II. (2017). Structure of IP(3)R channel: high-resolution insights from cryo-EM. Curr. Opin. Struct. Biol. 46, 38–47. 10.1016/j.sbi.2017.05.014. PMC5683905 28618351

[48] Schmitz EA, Takahashi H, and Karakas E. (2022). Structural basis for activation and gating of IP(3) receptors. Nat. Commun. 13, 1408. 10.1038/s41467-022-29073-2. PMC8930994 35301323

[49] Evans R, O’Neill M, Pritzel A, Antropova N, Senior A, Green T, Žídek A, Bates R, Blackwell S, Yim J, (2022). Protein complex prediction with AlphaFold-Multimer. Preprint at bioRxiv. 10.1101/2021.10.04.463034.

[50] Sattler M, Liang H, Nettesheim D, Meadows RP, Harlan JE, Eberstadt M, Yoon HS, Shuker SB, Chang BS, Minn AJ, (1997). Structure of Bcl-xL-Bak peptide complex: recognition between regulators of apoptosis. Science 275, 983–986. 10.1126/science.275.5302.983. 9020082

[51] Lock JT, Alzayady KJ, Yule DI, and Parker I. (2018). All three IP(3) receptor isoforms generate Ca^2+^ puffs that display similar characteristics. Sci. Signal. 11, eaau0344. 10.1126/scisignal.aau0344. PMC6402561 30563861

[52] Paillard M, Csordás G, Szanda G, Golenár T, Debattisti V, Bartok A, Wang N, Moffat C, Seifert EL, Spät A, and Hajnóczky G. (2017). Tissue-Specific Mitochondrial Decoding of Cytoplasmic Ca^2+^ Signals Is Controlled by the Stoichiometry of MICU1/2 and MCU. Cell Rep. 18, 2291–2300. 10.1016/j.celrep.2017.02.032. PMC5760244 28273446

[53] Rodríguez-Prados M, Berezhnaya E, Castromonte MT, Menezes-Filho SL, Paillard M, and Hajnóczky G. (2023). MICU1 occludes the mitochondrial calcium uniporter in divalent-free conditions. Proc. Natl. Acad. Sci. USA 120, e2218999120. 10.1073/pnas.2218999120. PMC10175726 37126688

[54] PEARC ’17 (2017). Practice and Experience in Advanced Research Computing 2017: Sustainability, Success and Impact (Association for Computing Machinery).

