# Supplementary material for: Intermembrane coupling between Bcl-xL and the IP3 receptor supports local Ca2+ transfer at ER-mitochondrial contacts
Source: Cell Rep. Author manuscript; Available in PMC 2026 Oct 9. (PMC13651792; doi:10.1016/j.celrep.2026.117767)
Supplement: Supplementary file 1 [file NIHMS2206900-supplement-1.pdf]

Cell Reports, Volume *45*

Supplemental information

Intermembrane coupling between Bcl-xL and the IP_3 receptor supports local Ca^{2+} transfer at ER-mitochondrial contacts

Arijita Ghosh, David Weaver, Chi Li, and György Hajnóczky

Supplementary Figures

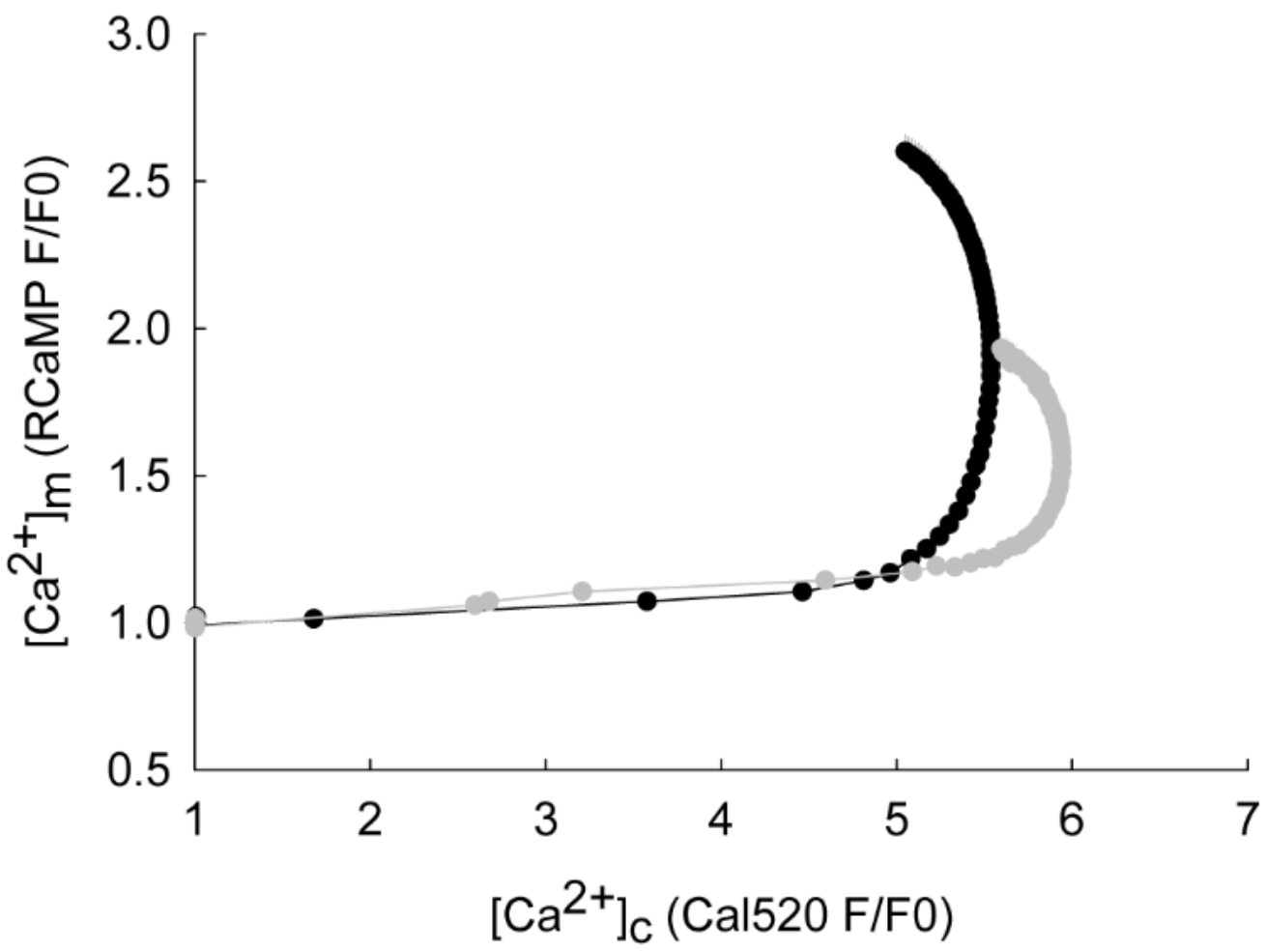

SFig1-Bcl-xL facilitates IP_3 induced IP3R mediated ER-mitochondria Ca^{2+} transfer. Representative traces showing IP3 induced $[Ca^{2+}]_m$ signals plotted as a function of $[Ca^{2+}]_c$ signals in WT (n=47) and Bcl-xLKO (n=60) MEFs showing earlier onset of $[Ca^{2+}]$m signal in WT with corresponding increase in $[Ca^{2+}]_c$ signals.

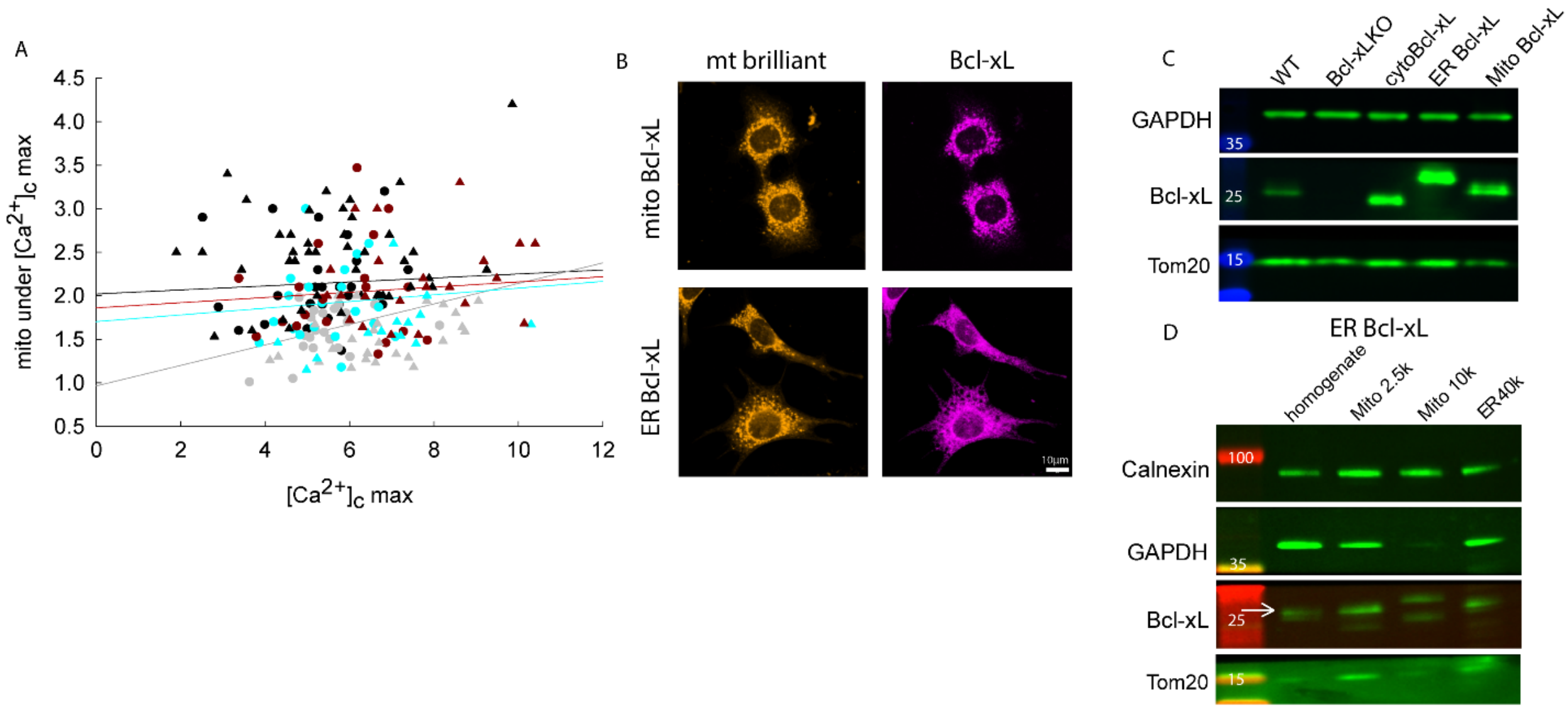

SFig2–Bcl-xL localization and effect on ER-mitochondria Ca^{2+} transfer. A. Scatter plots of $[Ca^{2+}]_m$ peaks or maximum $[Ca^{2+}]_m$ signal plotted as a function of $[Ca^{2+}]_c$ peaks or maximum $[Ca^{2+}]_c$ evoked by IP3 in WT n=67, Bcl-xLKO n=47, acute rescue n=33 and mitoBcl-xL stably rescued cells n=41 coming from N=2 independent experiments. B. Immunofluorescence images of MEFs expressing Mito Bcl-xL (upper panel) and ER Bcl-xL (lower panel), stained with MitoBrilliant646 as mitochondria marker. C. Western blot validating expression of Bcl-xL in the different organelle-targeted MEF models. D. Western blot images of sub-cellular fractions of ER Bcl-xL MEFs. Proteins used as markers are calnexin (ER), GAPDH (cytosol) and Tom20 (mitochondria).

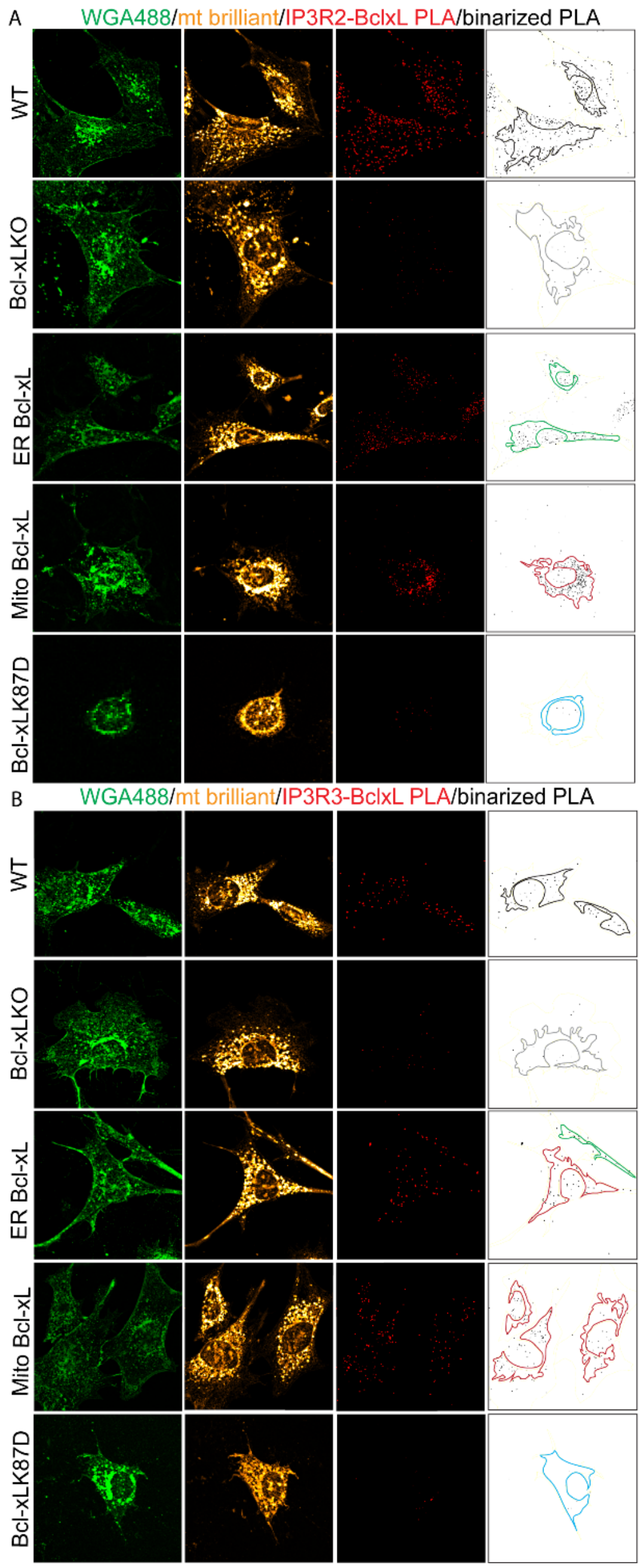

C

Bcl-xL	Condition (in MEFs)	IP3R	PLA mito
WT	WT	IP3R2	19.6±1.7
		IP3R3	7.5±0.75
KO	KO	IP3R2	6.6±0.57
		IP3R3	6.6±1.09
ER	Stable rescue	IP3R2	29.8±1.8
		IP3R3	26±4.9
Mito	Stable rescue	IP3R2	34.9±2
		IP3R3	19.7±1.95
K87D	Acute rescue	IP3R2	2.78±0.5
		IP3R3	0.94±0.2
KO	KO	IP3R2	3.38±0.5
		IP3R3	1.13±0.2

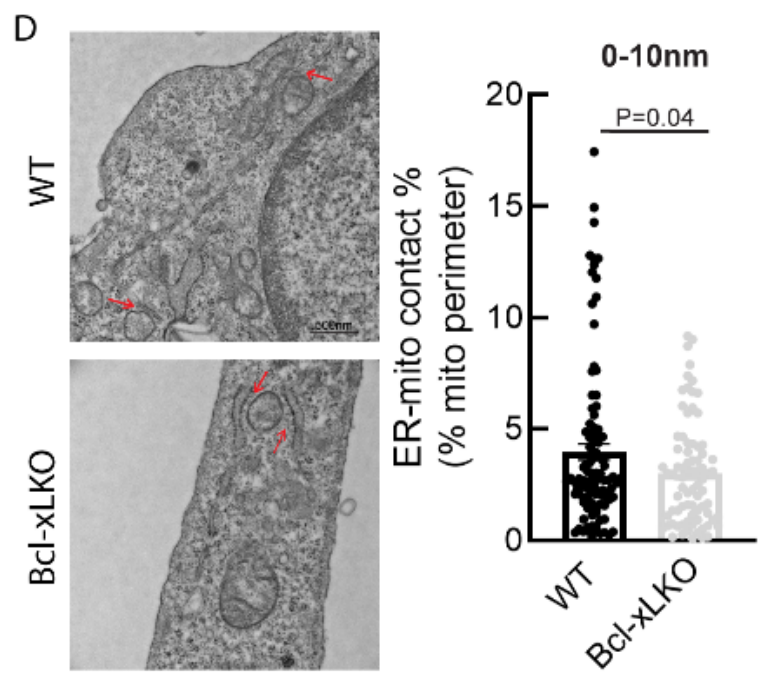

SFig3–Interaction between organelle targeted Bcl-xL and IP3R and impact of Bcl-xL on ERMC. A, B. Representative fixed (methanol) cell images from proximity ligation assay executed in MEFs to check interaction between endogenous IP3R2/IP3R3 and wild-type Bcl-xL or organelle targeted Bcl-xL using Bcl-xLKO and Bcl-xLK87D mutant as negative controls. These cells were stained with WGA488 and Mito Brilliant 646 to track the cell area and mitochondria (orange) respectively. C. PLA signal was quantified as density of PLA dots (no. per 100μm^2) calculated in entire cell (total) and mitochondrial regions (mito). The table describes the PLA signal obtained in the mitochondria masks using the IP3R isoforms in different conditions. D. Representative EM images for WT and Bcl-xLKO MEF cells showing ERMCs. Bar plots showing the percentage of mitochondrial perimeter within 0-10 nm distance to the ER in WT (black) and Bcl-xLKO (gray) MEFs. (WT n=101, Bcl-xLKO n=78 mitochondria from N=3 independent fixation; P=0.04 tested with unpaired t-test).

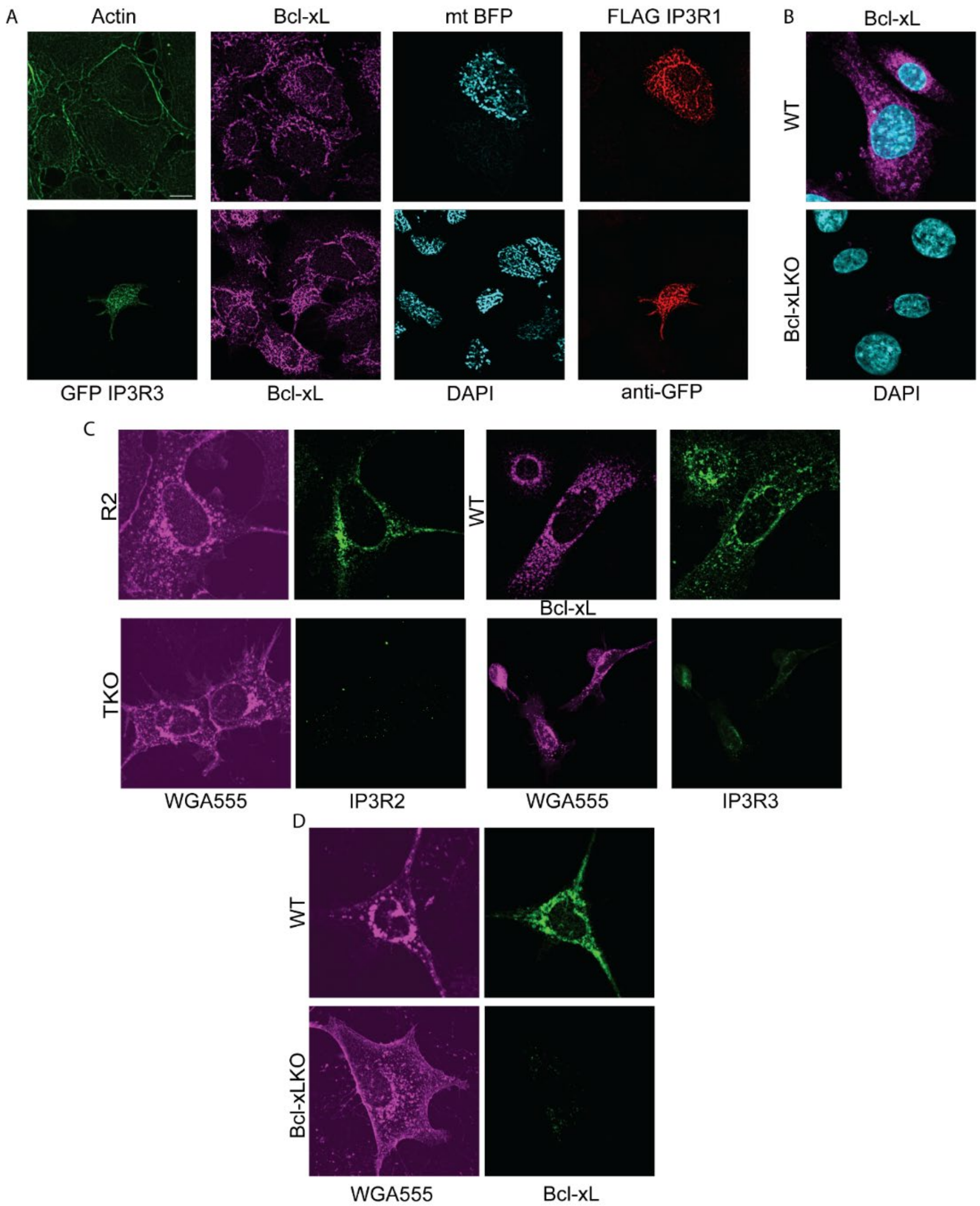

SFig4-Validation of primary antibodies used in PLA by immunocytochemistry. A. Representative PFA fixed images validating efficacy of antibodies used in PLA. In the upper panel TKO HEK cells co-transfected with IP3R1FLAG and mtBFP were stained with phalloidin to define cell boundaries. Primary antibodies against Bcl-xL and FLAG were detected with anti-rabbit568 and anti-mouse647 secondary antibodies respectively. In the lower panel, TKO HEK cells transfected with GFPIP3R3 were stained with DAPI to determine total number of cells in the field, GFP was used as a reporter for transfected cells. Primary antibodies against GFP and Bcl-xL were detected with anti-mouse647 and anti-rabbit568 secondary antibodies respectively. B. Representative images of PFA fixed WT and Bcl-xLKO mouse embryonic fibroblast cells stained with DAPI and primary antibody against endogenous Bcl-xL followed by anti-rabbit568 secondary antibody showing presence of Bcl-xL in WT only. C. Representative images of methanol fixed IP3R2 stable and TKO HEK cells stained with primary antibody against IP3R2 followed by anti-mouse488 secondary antibody validating IP3R2 antibody with no detection in TKO cells. Representative methanol fixed images of WT and Bcl-xLKO MEFs revalidated Bcl-xL antibody showing its presence in WT only and compatibility with methanol fixation.

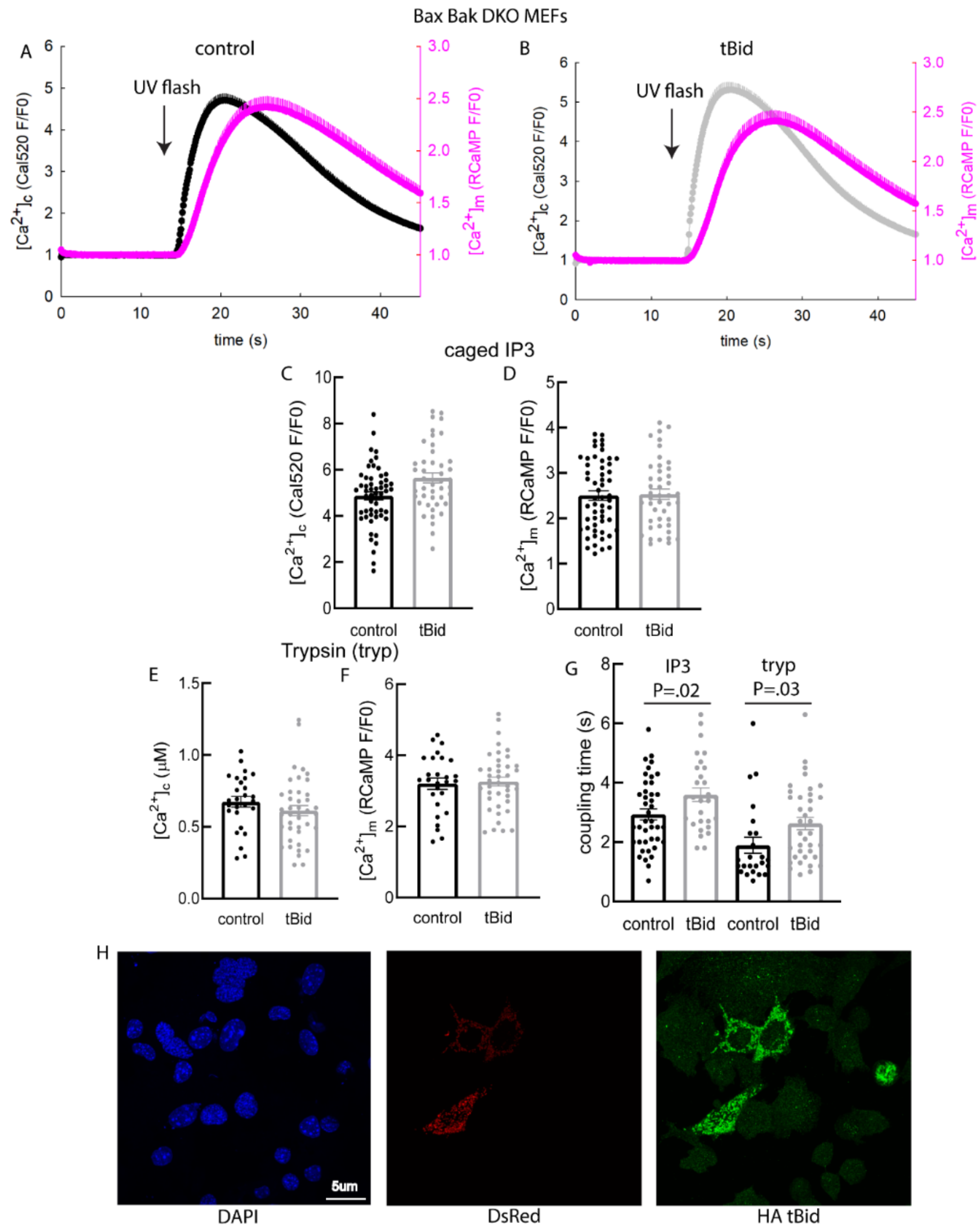

I

ICC	Total #of cells±SE	Only DsRed+	Only HA+	DsRed+HA
Bax Bak DKO MEF	122±15	2±0.6	1±0.8	7±2
IP3R TKO HEK	13±4	0.5±0.3	3±0.8	7±2

SFig5-Testing BH3 domain binding competence of tBid with Bcl-xL in Bax Bak double KO cells. A. Average traces of IP3 evoked simultaneous $[Ca^{2+}]_c$ and $[Ca^{2+}]_m$ signals in Bax Bak double knockout (DKO) MEF control cells (n=55) expressing $[Ca^{2+}]_m$ sensor RCaMP alone. B. Representative traces showing IP3 induced $[Ca^{2+}]_c$ and $[Ca^{2+}]_m$ signals in DKO MEFs (n=44) co-expressing tBid and RCaMP. C. IP3 evoked $[Ca^{2+}]_c$ peaks, significantly higher in tBid transfected cells compared to control. D. $[Ca^{2+}]_m$ peaks in control and tBid expressing cells show nearly equal $[Ca^{2+}]_m$ signals. E, F Trypsin (tryp) induced $[Ca^{2+}]_c$ and $[Ca^{2+}]_m$ peaks in control (n=27) and tBid transfected MEFs (n=41) respectively. G. Coupling time analysis shows longer coupling time in the case of tBid under both IP3 and trypsin treatment. H. Immunocytochemistry showing DAPI stained DKO MEFs expressing DsRed as reporter and HA tagged tBid co-expressing cells. I. Number of cells±S.E shown in the table belonging to the groups of DKO MEF and IP3R TKO HEK cells expressing DsRed or HA tagged tBid or both coming from n=3 fields.

A B

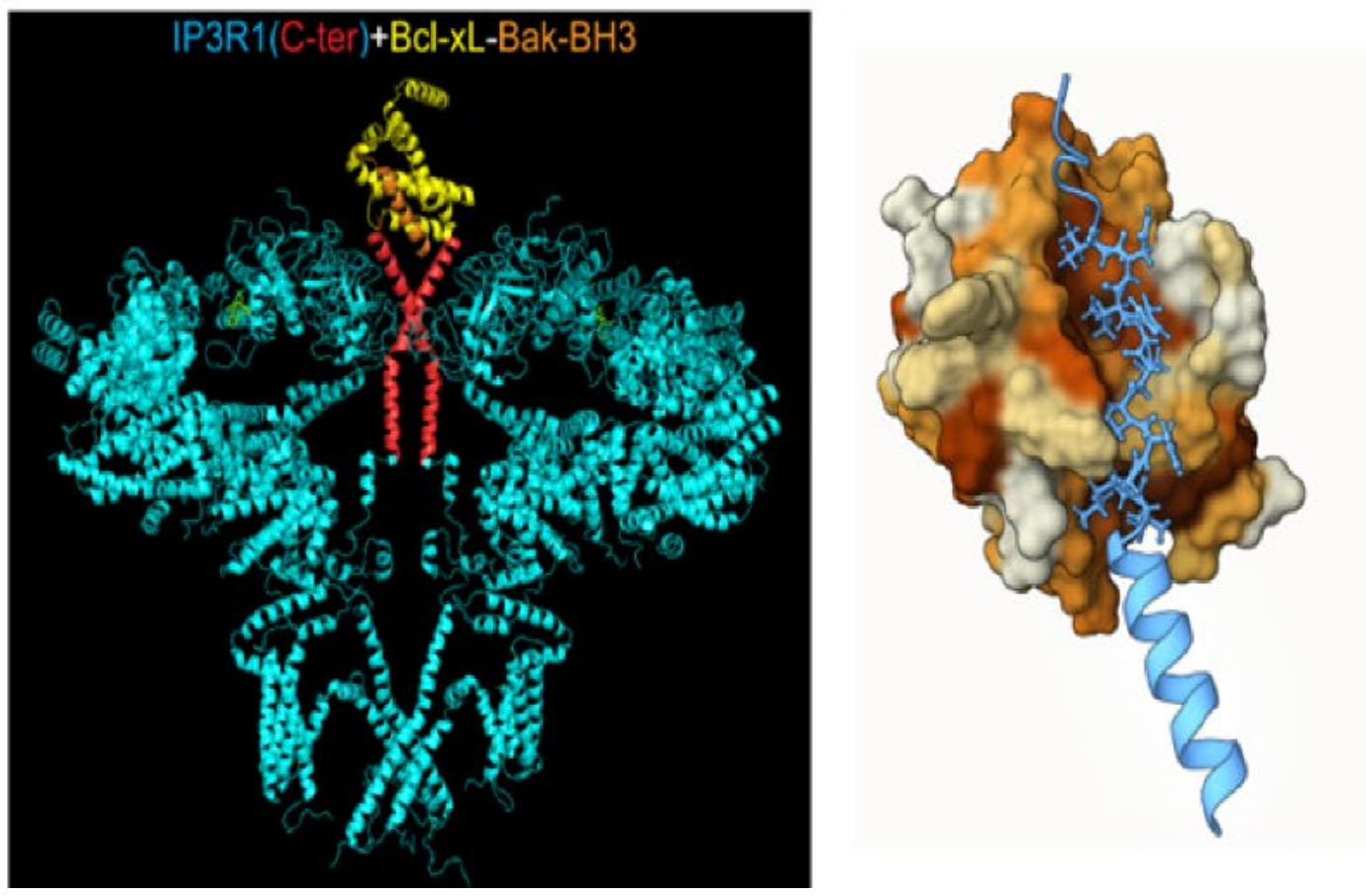

C

List of Bcl-xL residues interacting with R1 helix	Bcl-xL residues involved with BAK-BH3	Interaction type	BAK-BH3 partner residues
Ala93	Phe97	Hydrophobic	Val74
Phe97	Tyr101		
Tyr101	Leu108		
Leu112	Glu96	Hydrophobic	Leu78
Leu130	Phe105		
Trp137	Val141		
Arg139	Ala142		
Val141	Ala104	Hydrophobic	Ile81
Ala142	Leu130		
	Leu137		
	Arg139	Salt bridge /Electrostatic	Asp83
	Trp137	Hydrophobic	Ile85
	Phe146		

SFig6–*In silico* structural modeling of IP3R1 and Bcl-xL. A. Rendering of the 3D structures of human Bcl-xL in complex with the Bak-BH3 helix (yellow and orange, respectively; pdb_00005fmk) and ligand-bound rat IP3R1 (cyan, pdb_00006mu1) with the last C-terminal helix (C4) highlighted in red. Only two opposing IP3R molecules of the tetramer are shown. The ER-membrane traversing segments are at the bottom. B. Rendering of AlphaFold2 multimer predictions of the structure of mouse Bcl-xL [51](shown as the molecular surface with darker brown representing hydrophobic areas) in complex with the C-terminal flexible domain of mouse IP3R1 (cyan). The interacting segment is shown as ball & stick; the helical segment corresponds to the top end of C4 in (A). C. List of residues in predicted interface of Bcl-xL involved with IP3R1(Cter) and Bak-BH3 and its counter partners with the overlapping residues highlighted. Majority of the interactions are predicted in hydrophobic groove of Bcl-xL which is also crucial for binding BH3 mimetics.
